# Integrated Symbiotic Pleiotropy: Long Non-Coding RNAs and Disordered Proteins Interweaving the Functional Layers of the Eukaryotic Cell

**DOI:** 10.3390/ijms27083478

**Published:** 2026-04-13

**Authors:** Evelina Daskalova, Joon Seon Lee, Gergana Zahmanova, Ivan Minkov

**Affiliations:** 1 Department of Molecular Biology, University of Plovdiv, 4000 Plovdiv, Bulgaria; eve_das@uni-plovdiv.bg; 2 Faculty of Sciences, Brigham Young University–Hawaii, Laie, HI 96762, USA; joon.lee@byuh.edu; 3 Center of Plant Systems Biology and Biotechnology, 4000 Plovdiv, Bulgaria; 4 Institute of Molecular Biology and Biotechnologies, 4108 Markovo, Bulgaria

**Keywords:** integrated symbiotic pleiotropy (ISP), eukaryotic evolution, long non-coding RNA (lncRNAs), RNA–protein complexes (RNPs), membraneless organelles (MLOs), intrinsically disordered proteins (IDPs), molecular exaptation, telomerase reverse transcriptase (TERT), recombination activating gene 1 (RAG1), activity-regulated cytoskeleton-associated protein (Arc), symbiosis-driven evolution

## Abstract

Long non-coding RNAs (lncRNAs) and RNA–protein complexes (RNPs) are increasingly recognized as central to the regulatory complexity of modern eukaryotes. This review proposes that the remarkable diversity of eukaryotic systems arises from the long-term integration of ancient RNA/RNP mechanisms, layered with innovations introduced by successive symbioses. We outline four interconnected levels of symbiosis contributing to this process: (1) molecular symbiosis, involving dynamic assemblies of RNAs, proteins, and membraneless organelles (MLOs); (2) genome symbiosis, driven by the expansion of non-coding and repetitive DNA; (3) intracellular symbiosis, initiated by mitochondria acquisition; and (4) intercellular symbiosis, rooted in the cellular cooperation that enables multicellularity. We highlight lncRNAs and intrinsically disordered proteins (IDPs) as versatile mediators that interweave interactions across scales, predominantly within phase-separated condensates. Building upon these multi-level processes, we propose the framework of integrated symbiotic pleiotropy—a concept where molecular components acquire layered functional roles as a direct consequence of successive symbiotic acquisitions. This paradigm unites information layering, functional moonlighting, molecular tinkering, and exaptation into a coherent trajectory for eukaryotic evolution.

## 1. Introduction

The term “symbiosis,” originally defined in 1879 by A. de Bary as “living together of dissimilarly named organisms”, has since been debated, reinterpreted, and broadened [1,2]. Once limited to interactions between species, it now encompasses endosymbiosis and is widely used to frame phenomena from molecular interactions to genome evolution and multicellularity. At the molecular scale, Lanier et al. advanced the idea of “molecules in mutualism”, emphasizing reciprocal dependencies between cellular RNAs and proteins [3]. Since the 1980s, ecological metaphors—symbiosis, conflict, cooperation, and population dynamics—have been applied to the relationships between genomes and “selfish” genetic elements [4]. Multicellular organisms are often viewed as symbiotic communities formed through complex interactions among cells as members of a cooperative society [3,4,5,6].

It is widely accepted that RNAs and RNA–protein complexes (RNPs) were foundational biomolecules at life’s origin [7,8,9,10,11]. It is widely proposed that prebiotic RNA-centered molecular symbioses led to RNA–protein and RNP–lipid “worlds”, establishing the structural basis of life [12,13,14,15]. Modern omics analyses confirm that RNA/RNP mechanisms remain central to modern eukaryotic cells, underpinning complex subsystems and processes [11,13,16,17,18,19,20,21,22]. Specifically, long non-coding RNAs (lncRNAs) and intrinsically disordered proteins (IDPs) typically collaborate across scales—from discrete regulatory complexes to higher-order assemblies. Most notably, they drive the formation of self-organizing biomolecular condensates and membraneless organelles (MLOs), which provide a critical dimension to the global framework underpinning eukaryotic structural and operational complexity [23,24,25,26].

In this review, we define “genome symbioses” as the ancient and ongoing processes of conflict, co-evolution, and innovation transfer between proto-cells and virus-like entities. Such processes are presumably responsible for the emergence of the DNA genome—a provocative hypothesis of its viral origin [27] that stands alongside more traditional evolutionary scenarios, such as metabolic continuity and functional specialization [5,28,29]. These models and their integration into the framework of ISP are discussed in more detail in Section 3.

Over billions of years, two distinct trajectories emerged: prokaryotes (“hard-wired” organisms) adopted streamlined genomes with minimal RNA processing, whereas eukaryotes (“soft-wired” organisms) retained extensive RNA- and RNP-based processes. Eukaryotes developed repeat-rich genomes through the incorporation and exaptation of mobile elements [9,10,16,27,30] and harbor diverse “informational populations” whose ecological dynamics drive the evolution of biological complexity [31,32].

All these processes were further accelerated by mitochondrial endosymbiosis, which enhanced both molecular and genomic complexity [33,34,35,36], thereby facilitating the transition to multicellularity. The multifaceted impact of this symbiotic integration is explored in detail in Section 4.

**Figure 1:**
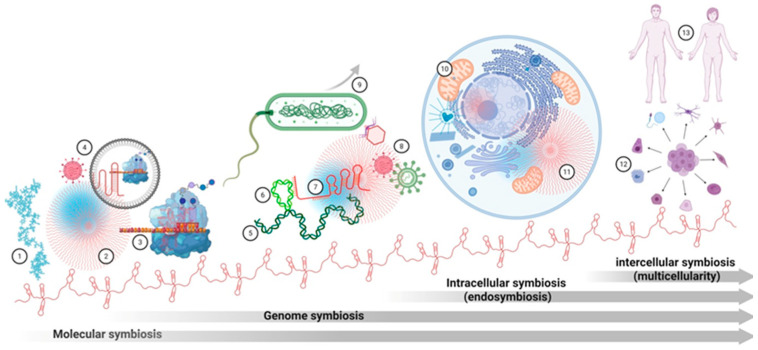
The eukaryotic journey from the RNA–protein world to complex multicellularity. Molecular symbiosis: (1) Intrinsically disordered proteins (IDPs) and (2) self-organizing ribonucleoprotein condensates (ribosoids). (3) The origin of protein translation as the first “organic code”. (4) Lipid encapsulation and the emergence of proto-cells (ribo-organisms) and proto-viruses. Genome symbiosis: (5) Emergence of the DNA genome and its associated enzymatic machinery. (6) Transposition and horizontal gene transfer. (7) Expansion of RNA–protein and RNA–DNA interactions, creating biomolecular condensates and RNA-based “social networks”. (8) Gene transfer agents (GTAs). (9) Prokaryotic divergence toward a streamlined, “hard-wired” lifestyle with reduced RNA-related processes. Intracellular symbiosis: (10) Mitochondrial endosymbiosis provides the energetic foundation for the expansion of the eukaryotic transcriptome, proteome, and signaling interactome. (11) MLO renaissance: the massive expansion of membraneless organelles in the nucleus and cytoplasm. Intercellular symbiosis: (12, 13) Emergence of epigenetic memory, cell differentiation, and intricate signaling networks, culminating in the development of complex body plans and multicellularity. Created in BioRender Zahmanova, G. (https://BioRender.com/wftne57, accessed on 5 April 2026) is licensed under CC BY 4.0.

The multi-level symbiosis probably fostered the emergence of new “organic codes” that underpin the rise of cellular and developmental complexity [37,38,39]. Here, we present a systems-level synthesis that links phenomena from life’s origins to modern multicellularity. These transitions are symbolically illustrated in Figure 1.

The four levels of symbiosis can be united within a conceptual framework we term integrated symbiotic pleiotropy (ISP). Within this framework, complexity is not merely an accidental accumulation of features but rather a coordinated interweaving of symbiotic legacies. Central to this process is the role of lncRNAs and IDPs, which—often acting within phase-separated environments—provide the cell with the necessary information density and functional flexibility. This molecular “fuzziness” allows a single entity to host multiple, overlapping programs, effectively transitioning the cell from a linear machine into a multi-layered, information-rich system, where the legacies of symbiotic acquisitions are preserved, repurposed and combined.

Although many lncRNAs and IDPs remain to be fully characterized, rapid advances in omics and structural modeling—including ribosome profiling, CLIP-seq, cross-linking MS, and AI-driven predictions—are steadily closing this gap [40,41,42]. By integrating ancient molecular origins with the systemic complexity of modern eukaryotes, our model suggests that lncRNAs and IDPs, whether as discrete factors or within biomolecular condensates, function as central organizers of cellular dynamics, signaling, and epigenetic regulation.

## 2. Molecular Symbioses: The Foundation of Biological Complexity

The principles of mutualism, traditionally applied to macroscopic ecosystems, have been proposed as a conceptual lens for understanding interactions at the biopolymer level. Lanier et al. [3] theorized that the “RNA makes protein–protein makes RNA” cycle represents a fundamental molecular mutualism, characterized by functional interdependence, co-evolution, and systemic robustness. Building upon these theoretical foundations, we expand this framework to trace how RNA–protein symbioses may have transitioned from prebiotic assemblies into the complex informational and operational networks of modern multicellular life.

### 2.1. RNA: The “Social” Molecule

Unlike the structural rigidity of DNA, RNA is a biochemically versatile and dynamic molecule. Despite its inherent instability, its capacity for transient, non-covalent interactions with proteins, nucleic acids, lipids, and metabolites (e.g., riboswitches) renders it a primary mediator of molecular networking [43,44]. This interactive versatility lies at the core of what we term “molecular symbiosis”—a concept that builds upon the “chemical symbiosis” proposed for an RNP-centered origin of life [15,45,46,47,48,49,50,51].

The RNP world hypothesis offers a potential resolution to the “chicken-or-egg” paradox by suggesting that life emerged from co-evolving RNA–protein assemblies rather than isolated polymers [5,15,16,51,52]. Within this framework, such mutualism is hypothesized to predate the first cells [15,27]. Modern eukaryotic RNP infrastructure probably represents an evolutionary extension of this ancient world [13], albeit with a significant functional transition. While ancestral RNAs likely focused on ribozyme activity and primitive scaffolding [47,50], modern long non-coding RNAs—partnered with (predominantly) intrinsically disordered proteins—have driven what has been described as an “RNP Renaissance” [12]. This partnership has significantly expanded the eukaryotic toolkit for sophisticated information processing, signaling, and regulation.

### 2.2. The Long Non-Coding Way

The regulatory capacity of RNA emerged at the dawn of evolutionary history. While in prokaryotes, lncRNAs are largely absent, diverse small regulatory RNAs (srRNAs), antisense RNAs, and CRISPR-associated RNAs play key roles, with functional parallels to eukaryotic lncRNAs such as effector guiding and macromolecular sequestration [53,54]. In eukaryotes, the non-coding transcriptome is typically categorized by length into small (<50 nt), intermediate (50–500 nt), and long non-coding RNAs (lncRNAs, >200 nt to >100 kb) [21,22,55].

LncRNAs can arise from diverse genomic loci: intergenic regions, introns, pseudogenes, repeats (satellite regions and transposons), or overlapping in any possible direction with protein-coding exons [11,32,56]. Remarkably, while protein-coding exons comprise only 1–2% of the human genome, up to 90% of it is transcribed into non-coding RNAs [22,57]. While critics caution against a “functional inflation” fueled by a potential publication bias, claiming that much of this pervasive transcription may represent RNA “junk” or “transcriptional noise” [58,59]—or highlight the challenges facing current in vivo functional assays [60]—a growing body of evidence suggests significant biological roles for a vast number of these transcripts. The disparity between ncRNA and protein-coding gene counts offers a plausible solution to the G-value paradox, suggesting that the expansion of the non-coding transcriptome—rather than the protein-coding gene count—drives the architectural complexity of multicellular life [11,57,61,62]. High-resolution transcriptomics now positions ncRNAs at the heart of cellular communication, from chromatin architecture to cytoplasmic signaling [19,20,22,40,63,64,65,66,67,68,69,70,71,72,73]. Consequently, it can be argued that ncRNAs serve as key architects of biological regulation across space and time [57,61,74,75].

Eukaryotic complexity is further amplified by bifunctional transcripts. On one hand, many mRNAs or their UTRs function as lncRNAs. While 5′UTRs often harbor hidden upstream Open Reading Frames (uORFs) and complex regulatory codes [76], 3′UTRs are increasingly recognized as sophisticated hubs that determine the fate and spatial localization of the transcript [77,78]. Empirical support for this informational density is found in transcripts where the mRNA size vastly exceeds the protein-coding requirements. Notably, certain transcripts encoding intrinsically disordered proteins (IDPs) possess unusually long mRNAs. For instance, the mRNA for MECP2 (transcript variant 1, NM_004992.4) spans over 10 kb to encode a relatively small protein with 86% experimentally assessed disorder (DisProt, [79]). Similarly, the adaptor protein GAB1 (NM_002039.5) is encoded by a nearly 8 kb transcript for a 694 aa sequence, with over 80% disorder (DisProt). Such cases suggest a potential link between high intrinsic disorder and increased non-coding RNA territory, where vast untranslated segments likely function as complex, high-capacity regulatory platforms for integrating multiple cellular signals. On the other hand, the blurring of boundaries between “protein-coding” and “non-coding” RNAs suggests that the eukaryotic genome acts as an evolutionary reservoir for proto-genes—sequences with nascent coding potential—and microgenes that encode functional micropeptides [40,80,81,82,83,84,85,86]. This double functionality can be regarded as “moonlighting” at the RNA level. It aligns with the concept of overlapping codes proposed by Trifonov [87], where a single sequence carries multiple layers of information, and directly supports our ISP framework. We propose that lncRNAs represent a highly versatile form of genetic coding. By not being restricted to a single functional output, they can evolve a dense architecture of overlapping signals—either as “pure” regulatory molecules or as bifunctional transcripts. This multi-layered information density enables the eukaryotic genome to achieve unprecedented levels of integration, where a single locus serves multiple interrelated symbiotic roles.

Crucially, this RNA-based complexity is underpinned by a deep symbiosis with protein partners. Unlike “classical” RNA-binding proteins (RBPs) that rely on structured motifs (e.g., RRM, dsRBM, and KH), the RNA-binding proteome is highly enriched in intrinsically disordered regions (IDRs) [88]. This lack of fixed structure allows for the dynamic, multivalent interactions that underpin the complexity of modern cellular life [89,90].

### 2.3. On the Edge of Chaos: Functional Properties of IDPs

The classical “sequence–structure–function” paradigm [91,92] has been fundamentally revised by the discovery of intrinsically disordered proteins (IDPs). Lacking a stable tertiary fold, these molecules exist in a highly dynamic state—often described as being “on the edge of chaos”—fluctuating within a conformational ensemble of multiple metastable states [92,93,94,95]. Paradoxically, this lack of rigidity enables their diverse cellular roles, from transcriptional regulation to signal transduction [95,96,97]. Their conformational plasticity allows a single disordered region to act as an interaction hub, engaging hundreds of different partners to rewire cellular networks in response to internal and external cues [98,99,100,101]. In this sense, protein disorder can be viewed as a structural isomorphism to genetic code degeneracy; just as multiple codons can map to a single amino acid, a single disordered sequence maps to a vast ensemble of functional states, enabling the integration of diverse signals and partners.

IDPs are evolutionarily ancient and likely played a key role in the origin of life. Prebiotic polypeptides, such as glycine-alanine tracts, were presumably highly disordered and capable of spontaneous self-demixing and self-organization—a foundational property for the assembly of modern membraneless organelles (MLOs) [100].

Proteins are typically categorized by their proportion of disordered residues: ordered (0–10%), moderately disordered (10–30%), and highly disordered (>30%). While shorter segments usually act as flexible linkers between ordered domains, disordered regions longer than 30 amino acids often function as specialized molecular binding interfaces [97]. These intrinsically disordered regions (IDRs) are now recognized as essential for genome organization, stress response, and adaptation to extreme environments [100,101,102,103,104].

Despite the pervasive nature of intrinsic disorder, caution is warranted when inferring specific biological functions solely from sequence-based disorder predictions [105]. Critics argue that in many cases, disordered regions may simply serve as flexible linkers or remain biochemically inert under physiological conditions. The significant challenge remains in bridging the gap between bioinformatic inference and experimental validation, as the dynamic nature of IDPs often escapes traditional structural biology techniques, leading to potential overestimations of their regulatory impact in complex cellular environments [105,106].

Notably, IDPs are present across all kingdoms of life, including viruses [98,99]. Mirroring the trend observed for lncRNAs, protein disorder scales with organismal complexity. While IDPs constitute ~2% of the proteome in archaea and 4% in Eubacteria, they reach up to 44% in the human proteome [100,101]. Although this trend is not strictly linear [102], IDPs have reached unprecedented diversity in eukaryotes, where they orchestrate complex functions in signaling, transport, and biomolecular condensate formation [101,107].

This synchronized expansion of the disordered proteome and the non-coding transcriptome suggests a profound co-evolutionary synergy [57,61,90]. In this model, ordered RNA-binding domains provide sequence specificity, while IDRs recognize the complex structural scaffolds inherent in lncRNAs. By engaging lncRNAs and other targets, IDRs provide a multivalent framework for the assembly of biomolecular condensates [90]. Consequently, lncRNA–IDP interactions emerge as a significant component of eukaryotic spatial organization [88,90,108], complementing traditional membrane-bound compartmentalization to achieve higher-level cellular integration.

### 2.4. Liquid Architectures: The Self-Organizing World of Membraneless Organelles

As highly effective binders, IDPs often form “fuzzy complexes”—dynamic, conformationally unstable assemblies that allow for the simultaneous sampling of multiple interaction states [69,109]. Their capacity for multivalent interactions inherently favors a high aggregation propensity; specifically, prion-like disordered domains function as “molecular stickers”, promoting liquid–liquid phase separation (LLPS) in crowded cellular environments. Parallel to this, RNA—and lncRNA in particular—is recognized for its dynamic architectural role in these biomolecular condensates [108]. LLPS drives the spontaneous formation of droplets or coacervates—structures lacking lipid membranes but defined by sharp phase boundaries formed by abrupt shifts in density and concentration between the condensed phase and the surrounding dilute environment [24,26].

The discovery of phase-separated assemblies is widely regarded as a new frontier in cell biology, potentially redefining the classical view of the cytoplasm [26,110]. However, critics warn that LLPS is often invoked without sufficient in vivo evidence, potentially oversimplifying the complex, non-equilibrium nature of the cytoplasm and nucleus. Rather than being a universal panacea, phase separation in the cell is often tightly regulated and constrained by stable structural scaffolds. More generally, its prevalence may be overstated at the expense of stereo-specific molecular interactions and active, energy-driven processes [111,112].

The concept of phase-separated condensates predates the discovery of IDPs and MLOs. In early abiogenesis theories, Haldane and Oparin proposed coacervates as precursors of life [113,114], an idea compatible with RNP world scenarios. Similarly, Barbieri’s ribotype theory suggests that ancient ribonucleoprotein particles, or “ribosoids”, were the fundamental units of life, with the ribosome as the central ribosoid of the first organic code [37,38,39,50]. Modern MLOs share defining features with these ancient ribosoids: self-organization, reversible assembly, selective compartmentalization, and the capacity to bridge molecular and cellular scales [37,38]. In both ancient and contemporary systems, IDPs and lncRNAs are the recurrent essential components [88,115]. Figure 1 (“Molecular symbiosis” section) symbolically illustrates the key players in the molecular transitions between the ancient and modern RNP worlds.

Recent evidence suggests that the hallmarks of eukaryotic complexity—non-coding RNAs, IDPs and MLOs—exist in prokaryotes in simpler, functionally relevant forms [116,117]. While bacteria and archaea largely lack lncRNA, their MLOs are predominantly protein-based [116]. A well-characterized example is the BR-body in *Caulobacter crescentus*, which assembles on the intrinsically disordered domain of RNAse E and functionally resembles eukaryotic stress granules [117,118]. Furthermore, many bacteria form stress-induced aggresomes via LLPS to enhance survival during starvation or antibiotic treatment [118]. This inherent stress-responsiveness of bio-condensates appears to be an ancient, conserved feature across all domains of life [119]. Representative examples of these eukaryotic MLOs and their convergent prokaryotic counterparts, together with the disorder content of their marker proteins, are summarized in Table 1.

Together, these observations support dynamic compartmentalization via phase separation as an evolutionarily ancient strategy predating eukaryotes. Subsequent diversification likely reflects the refinement of these systems rather than the invention of entirely new mechanisms [130]. Across all domains, condensates have convergently evolved as energy-efficient, biochemically distinct compartments [130,131]. This is most pronounced in eukaryotes, where over 100 identified MLO types exhibit remarkable mobility and reversible assembly across the nucleus, cytoplasm, and endosymbiotic organelles [24,25,26,122,123,124,125,126,127,128,129]. Hallmark examples of MLOs across eukaryotes, prokaryotes, and organelles are presented in Table 1; for further technical specifications and isoform identification, see Supplementary Table S1.

Since most eukaryotic MLOs are composed of RNA and IDR-rich proteins, the lncRNA–IDP interaction represents a universal principle of MLO genesis and function [107,108]. Despite the numerical superiority of proteins (up to 10^7^ copies/cell) over scarce lncRNAs (0.3–1000 molecules/cell) [132], lncRNAs exert a disproportionate influence on MLO behavior through several key mechanisms: [107,108,132,133,134]:

- Multivalent scaffolding: Facilitating weak, transient interactions via a negatively charged backbone.
- Structural plasticity: Offering diverse secondary structures and modifications that provide functional versatility.
- Spatial seeding: Guiding MLO nucleation through precise base-pairing.
- Stoichiometric buffering: Preventing aberrant protein aggregation by maintaining optimal RNA-to-protein ratios.

In the ISP framework, the emergence of liquid-like condensates represents a systemic isomorphism that reflects, at the cellular scale, the high informational density of the genome. Just as overlapping codes compress multiple regulatory layers into a single sequence, the multivalent nature of IDPs and the functional versatility of moonlighting proteins mirror this economy of scale. In turn, biomolecular condensates provide the necessary fluid, multi-state architecture to integrate these complex, interleaved signals into a coherent regulatory output.

This multi-scale plasticity allows the eukaryotic cell to transcend the “one-gene, one-structure, one-function” dogma, shifting the focus from discrete components to an integrated symbiotic continuum. Through these mechanisms, IDPs and lncRNAs bridge molecular interactions with cellular architecture, serving as spatiotemporal hubs that remain ultimately anchored in the stable, transgenerational blueprint of DNA.

## 3. Genome Symbioses: DNA as the Eternal Library of Life

### 3.1. The Evolutionary Transition to the DNA Genome

The hypothesis of an ancestral RNA-based genome remains the prevailing paradigm in evolutionary biology, providing the necessary context for exploring more complex genomic transitions [135]. The emergence of the DNA-based genome is traditionally described by several complementary models that address the chemical and informational constraints of early life. The metabolic shift hypothesis (Lazcano’s model) emphasizes that DNA was selected as a late, chemically stable archive to protect accumulated genetic complexity from hydrolytic and UV damage [28]. This is further supported by the methyl-RNA bridge concept, which proposes an intermediate phase where 2′-O-methylation of RNA provides the structural stability necessary to overcome the Eigen limit—the informational bottleneck (approx. 30 kb) beyond which error-prone RNA replication leads to mutational meltdown [29].

A fundamental theoretical framework for this shift was provided by Maynard Smith and Szathmáry (1995) in their analysis of major evolutionary transitions [5]. They characterized the emergence of the DNA-protein world as a critical division of labor: DNA became specialized for high-fidelity, long-term information storage, while proteins took over the vast majority of catalytic tasks. In this “standard model,” RNA was relegated to a transient intermediary (mRNA) or a structural scaffold (rRNA), effectively decoupling the digital information (genotype) from the functional execution (phenotype) [5].

However, a more transformative perspective—in line with the ISP framework—is provided by Patrick Forterre’s viral invention hypothesis [27,136,137]. Forterre suggests that DNA was a late evolutionary innovation, emerging not within cells, but within the virosphere as a protective adaptation against host RNA-degrading enzymes. [27,138,139]. In this model, the Last Universal Common Ancestor (LUCA) was a cellular “ribo-organism” with an RNA genome and proto-ribosomes capable of translation, enclosed within a lipid membrane. These “RNA cells” co-existed with RNA- and DNA-based proto-viruses, which likely emerged from RNA cells through the parasitic reduction in their ancestral translation machinery [27,137].

The transition to the DNA genome was, in effect, a colonization process. Viral DNA genomes initially co-existed with cellular RNA as plasmids before losing their capsid genes and becoming resident, non-infectious retroelements, such as endogenous retroviruses (ERVs) and transposons, marking a shift toward a more stable deoxy form of RNA [139,140]. Forterre proposes that DNA originated in RNA viruses as a protective adaptation against host RNA-degrading enzymes [137,141]. Supposedly, these viral DNA genomes eventually colonized RNA cells, initially co-existing as plasmids before losing their capsid genes to become resident, non-infectious retroelements (ERVs and other transposons [142,143,144]). Over time, DNA displaced RNA as the primary carrier of genetic information [139]. This transition was mediated by the reverse transcriptase (RT), an ancient enzyme whose distant homology with all modern polymerases supports the view of retroelements as the universal ancestral units of DNA genomes [145,146].

The adoption of DNA is further supported by the shift from a single ancestral enzyme (RdRp) to specialized polymerases for transcription (DdRp) and replication (DdDp) [147]. Notably, the existence of three non-homologous families of replicative DNA polymerases (PolC in bacteria, PolD in archaea, and PolB in eukaryotes) confirms that replication mechanisms evolved independently and late across the three domains of life [148]. Thus, the eukaryotic genome is best viewed as an ongoing process of mutual exchange and co-existence—a genomic symbiosis still active in modern eukaryotic cells [144].

### 3.2. Genomic Sociology: Conflicts, Cooperations, and Exaptation

While the classical view of the genome emphasizes vertical inheritance and individual gene fitness, an emerging conceptual framework—often termed genomic sociology—proposes a more collective, agent-based perspective. Although partly speculative, this model is increasingly supported by comparative genomics and molecular evidence, viewing the genome not as a static blueprint but as a dynamic collective of competing, cooperating, and communicating agents.

Complexity through defense: As Eugene Koonin describes in The Logic of Chance, genomic complexity can be viewed as a byproduct of the perpetual “arms race” between hosts and parasites [149]. In this model, parasitic elements inevitably emerge, and host survival is possible only through the evolution of complexity—specifically through compartmentalization and sophisticated regulation. While parasites tend to simplify, hosts must become increasingly complex in response [149,150]. From this perspective, parasites—including viruses and transposable elements (TEs)—can be viewed as complexity-provoking symbionts. The ongoing pressure from these elements drives the evolution of host defense mechanisms, such as epigenetic silencing and recombination, which are eventually exapted into essential regulatory layers. Once dismissed as “selfish DNA,” these elements are now recognized for their vital co-evolutionary role in genome plasticity [142,143,144,151,152].

Molecular domestication: Through exaptation (or molecular domestication), the host cell repurposes infectious tools for internal functions. Ancient examples include gene transfer agents (GTAs)—virus-like particles used by prokaryotes to package host DNA [153,154]. In eukaryotes, hallmark examples of this synergy include telomerase reverse transcriptase (TERT), RAG1 recombinase, and the neuronal Arc protein, all of which originated from ancient transposable elements (detailed in Section 6).

Luis Villarreal and Guenter Witzany explicitly describe the collective of domesticated elements as an RNA-based communication system [31]. In their model, DNA serves as the stable “house” of genetic storage, while various RNAs (rRNAs, tRNAs, introns, retroelements, lncRNAs, etc.) form a “social network” governed by a “stem-loop language” [155,156].

Structuring the social genome: This social organization is fundamentally tied to the physical state of the genome. Growing evidence suggests that basic chromatin architecture and function are inseparable from RNA. Chromatin-associated RNAs (caRNAs) shape nuclear architecture and epigenetic regulation [39]. For instance, transcripts from constitutive heterochromatin (satellite RNAs and TERRA) maintain repressed states, while lncRNAs near imprinted regions regulate facultative heterochromatin [157]. Furthermore, L1-derived ncRNAs help maintain euchromatin, while diverse, unique, and repetitive RNAs organize the three-dimensional nuclear landscape via R-loops, triple helices, and biomolecular condensates such as MLOs [158,159,160,161]. As represented in the “Genome symbiosis” section of Figure 1, the interactions among genomes, transposons, GTAs, and non-coding RNAs—potentially mediated by biomolecular condensates—constitute a fundamental layer of eukaryotic complexity.

Repeats as a scaffold for phase separation: Recent models suggest that repetitive elements (satellite DNA, TEs) act as primary drivers of liquid–liquid phase separation (LLPS) due to their inherent multivalency. These repeats and their “sticky” lncRNA transcripts provide dense binding motifs that recruit IDR-rich proteins, nucleating biomolecular condensates and defining specific nuclear territories. This mechanism facilitates the formation of dense heterochromatin (e.g., HP1-mediated silencing) and sequesters mobile elements to maintain genomic stability [162].

Genomic streamlining vs. informational expansion: The strategies for biological complexity diverge sharply between domains. Prokaryotes operate under intense selective pressure for metabolic efficiency and rapid replication, leading to genomic streamlining. Their evolution is a zero-sum balance of DNA gain and loss, perpetually recalibrating their genomes and proteomes to immediate environmental demands [10,36]. In contrast, eukaryotes function as information accumulators. Rather than purging foreign elements, they retain and frequently exapt GTAs, retrotransposons, and viral remnants as “living libraries” for regulatory and structural innovation [163,164,165].

### 3.3. Synthesis: ISP and the Genome Sociology

This remarkable RNA-based complexity could only flourish once the DNA genome became stabilized, providing an anchor for the long-term evolutionary memory. The DNA genome—the most intricate information system known—does not operate through a single blueprint. Instead, it functions through multiple, overlapping codes [37,39,87,166], reflecting the networking sociology of life’s origins. This multifaceted coding is best understood as a direct legacy of genomic symbiosis, where diverse evolutionary agents integrated their information into a single, functionally unified, yet modular system [87,166]. Synthesizing these multi-scale observations, we propose a fundamental systemic isomorphism between the degeneracy of the genetic code and the conformational multivalency of lncRNAs and IDPs. Just as the redundancy of the triplet code enables the layering of overlapping regulatory programs within the DNA—acting as a multi-dimensional archive—the intrinsic “fuzziness” of disordered proteins and non-coding RNAs provides a permissive, symbiosis-promoting landscape for functional innovation. This molecular plasticity ensures that the DNA genome can accommodate the “social” interactions of diverse symbiotic origins without disrupting its core integrity. In this view, the eukaryotic genome serves as a dynamic, layered record of its symbiotic past. This synergy defines the essence of integrated symbiotic pleiotropy (ISP), where molecular redundancy becomes the primary vehicle for systemic integration.

While the physical process of phase separation is an inherent, energy-efficient property found across all domains of life, its vast expansion in eukaryotes was catalyzed by mitochondrial endosymbiosis. This bioenergetic revolution provided the basis for a massive scale-up of cellular complexity, enabling a sophisticated compartmentalization through both canonical membrane-bound systems and dynamic, RNA-driven phase separations that define the modern eukaryotic cell.

## 4. Intracellular Symbiosis: The Endosymbiotic Transformation

### 4.1. An Energetic and Architectural Revolution

The acquisition of mitochondria is widely regarded as the most transformative event in cellular history [167,168,169,170]. Beyond providing an unprecedented energy surplus, the integration of the endosymbiont is proposed to have triggered a fundamental informational and structural remodeling of the host. Through the massive influx of bacterial genes and foreign lipids, this symbiotic merger provided the energy and materials necessary to construct the complex architecture of the eukaryotic cell [36].

This transition unlocked new dimensions of biological organization:

- Unprecedented energy abundance: The internalization of mitochondria resulted in a vast expansion in ATP production, creating an unprecedented “metabolic budget” that far exceeds any prokaryotic system. Estimates suggest that endosymbiosis expanded the bioenergetic capacity by four to five orders of magnitude per gene, providing approximately 1000 to 10,000 times more energy than that available to a typical bacterium [34,36,171]. This bioenergetic leap was the essential prerequisite for supporting the high cost of maintaining complex genomic and structural innovations.
- Genomic prosperity and chimeric complexity: This abundant energy allowed eukaryotes to sustain large genomes, rich in repetitive elements and non-coding RNAs. Endosymbiotic gene transfer (EGT) further amplified this by creating chimeric genomes, merging an archaeal “informational core” (flexible regulation) with a bacterial “operational system” (metabolic efficiency) [35,172].
- The intron invasion and the nucleus: It is hypothesized that the influx of bacterial group II introns from the endosymbiont necessitated the physical separation of transcription and translation, driving the formation of the nucleus as a defensive and regulatory barrier [33,173]. Under the nuclear “firewall,” the emerging intron–exon architecture facilitated widespread exon shuffling and alternative splicing, allowing for a complex, multi-domain proteome [174,175].
- Enhanced signaling and active behavior: Liberated from energy constraints, the plasma membrane evolved into a sophisticated sensory interface. This transition was marked by a crucial lipid divergence: while bacterial fatty acids provided membrane fluidity, ancient archaeal isoprenoid pathways were repurposed into specialized signaling molecules, such as steroids [36,176]. Combined with an expansive receptome, this molecular diversity allowed eukaryotes to develop the intricate signaling networks essential for complex life [177]. Furthermore, by overcoming the geometric surface-to-volume constraints that limit bacterial size, mitochondrial energy allowed eukaryotic cells to expand their volume. The plasma membrane, supported by the cytoskeleton, became a flexible tool for dynamic reshaping, enabling active behaviors, such as amoeboid movement and phagocytosis—essential elements of multicellular coordination [178].
- Vesicular dynamics and logistics: The integration of archaeal protein machineries (ESCRT and SNARE complexes) with fluid bacterial lipids revolutionized cellular logistics [179,180]. Both systems utilize intrinsically disordered regions (IDPs) to provide the mechanical energy required for membrane dynamics [181]. This “vesicular revolution” gave rise to the complex transport and logistics of the endomembrane system—the ER, Golgi apparatus, and an endless stream of trafficking vesicles [182]. Consequently, a hierarchical structuring was established where membrane-bound surfaces act as platforms for nucleation and regulation of MLOs (see Section 4.2).

In the modern cell, mitochondria remain active partners, governing cellular fate through the retrograde signaling axis. By utilizing a “language” of Ca^2+^ fluxes, reactive oxygen species (ROS), and mitokines, mitochondria communicate their metabolic status to the nucleus, balancing homeostasis with programmed cell death [183].

### 4.2. The Symbiosis of Membranes and Droplets

Phase transitions within mitochondria. Notably, liquid–liquid phase separation (LLPS) occurs even within the bioenergetic heart of the cell. Mitochondrial RNA granules (MRGs) serve as membraneless hubs for mitoribosome biogenesis, functionally mirroring the nucleolus and exhibiting hallmark liquid-like behaviors. These granules contain mitochondria-encoded ncRNAs, mtDNA, RNA-processing proteins, and mitoribosome assembly factors [127,184].

While the mitochondrial proteome is generally more ordered, key metabolic enzymes (including those of the Krebs cycle) possess disordered regions that promote condensate formation. Furthermore, the highly structured membranes of the cristae may serve as a physical template for phase separation, favoring the emergence and stabilization of mitochondrial MLOs [127,185].

Hybrid architecture as a legacy of the endosymbiosis. Inside the eukaryotic cell, a dynamic interplay unfolds between membrane-bound organelles (MOs) and membraneless organelles (MLOs). We propose that this architecture is a direct legacy of endosymbiosis: the expansion of the internal membrane system necessitated specialized protein machinery to manage these new spaces.

Recent models suggest that the orchestration of membrane dynamics is fundamentally driven by the phase behavior of IDPs. For example, SNARE proteins organize into liquid-like clusters to ensure rapid membrane fusion, while ESCRT subunits form condensates that exert the mechanical forces necessary for fission [181,186]. Another related phenomenon is membrane wetting, where the interfacial tension between a liquid droplet and a membrane surface can spontaneously induce membrane curvature, stabilize damaged membranes, or modulate lipid packing [187,188].

By integrating LLPS with membrane physics, the eukaryotic cell achieves a form of membrane-condensate symbiosis. This synergy transformed the cell from a minimalist survival machine into a complex system capable of organizing dynamic molecular superstructures. The mitochondrial endosymbiosis thus provided the bioenergetic and structural framework for this functional integration—an evolutionary shift captured in the “Intracellular symbiosis” section of Figure 1.

While all these nested symbioses shape the internal landscape of the cell, the hierarchy of integration extends far beyond individual boundaries. The emergence of multicellularity represents the next evolutionary milestone, where lncRNAs, IDPs, MLOs, and membranes evolve from internal organizers into architects of complex, integrated organisms.

## 5. Symbiosis Beyond the Cell: The Rise of Multicellularity

The emergence of complex multicellularity represents a central phase transition in biological organization: a shift from individual cell survival to the orchestration of a macroscopic biological collective. In this key evolutionary step, lncRNAs, IDPs, and biomolecular condensates appear to act as key organizing factors, rewiring a single genome into the diverse cellular identities required for complex life. While simple multicellularity exists across various lineages, including prokaryotes, the transition to complex multicellularity with its 3D macrostructures and body plans remains a uniquely eukaryotic phenomenon [189].

### 5.1. RNA as the Architect of Multicellularity

Simple multicellularity. The origins of RNA-based regulation predate metazoans. Unicellular relatives of animals, such as *Capsaspora owczarzaki*, already utilize lncRNAs for lifecycle transitions [190]. In social amoebae *(Dictyostelium* spp.), specific mid-sized ncRNAs are essential triggers for aggregative behavior [191], absent in strictly unicellular amoebae, despite these species sharing a remarkably similar proteome [191,192]. Similarly, the transition from the unicellular *Chlamydomonas* to the closely related colonial *Volvox carteri* is associated with an expansion of lncRNAs that facilitate cell-type differentiation and stress responses. This suggests that multicellularity often arises not from new protein-coding genes, but from the “rewiring” of existing genetic networks through novel RNA-based instructions [193]. In basal metazoans like the sea sponge *Amphimedon queenslandica*, thousands of developmentally regulated lncRNAs have been identified, indicating that lncRNA regulatory mechanisms were probably deeply integrated into the animal lineage before the divergence of Eumetazoa [194,195].

Complex multicellularity evolved independently in several clades [189], all of which appear to have adopted lncRNAs as flexible regulators to synchronize epigenetics, signaling and tissue homeostasis:

- Plants: Non-coding RNAs engage in a multi-layered symbiosis [196,197,198,199,200,201], channeling mobile DNA into regulatory innovation and coordinating organellar and nuclear genomes via retrograde signaling [202,203]. At the intercellular level, they travel through plasmodesmata and phloem to align stress responses across distant tissues [204,205]. Their extensive roles in *Arabidopsis* and other species are reviewed elsewhere [196,197,198,199,200,201,202,203,204,205].
- Fungi: Fungal systems utilize a “hidden genome” of antisense lncRNAs and exapted transposable elements to synchronize thousands of nuclei within shared syncytial networks [206,207,208,209,210,211]. Unique strategies, such as “Starships” (massive transposable platforms for horizontal gene transfer), drive rapid niche specialization by integrating mobile elements with lncRNA-driven plasticity [212].
- Animals: In metazoans, lncRNAs are proposed to stabilize tissue identity by coordinating enhancer functions and chromatin remodeling [210,211,213,214,215,216,217,218,219,220,221,222,223]. They are indispensable for body plan formation and embryo development, primarily through the regulation of homeobox (HOX) clusters [224,225,226,227,228]. In the nervous system, transcripts like *MALAT1* and the human-specific *HAR1A* guide synaptogenesis and neuronal migration [229], while other human-specific transcripts guide cortical development [229,230,231]. Ultimately, animal lncRNAs provide the stable yet flexible control necessary for cognitive complexity and organismal stability, while their dysregulation is a hallmark of senescence and disease [232,233,234,235,236,237,238,239,240,241,242,243].

**Figure 2:**
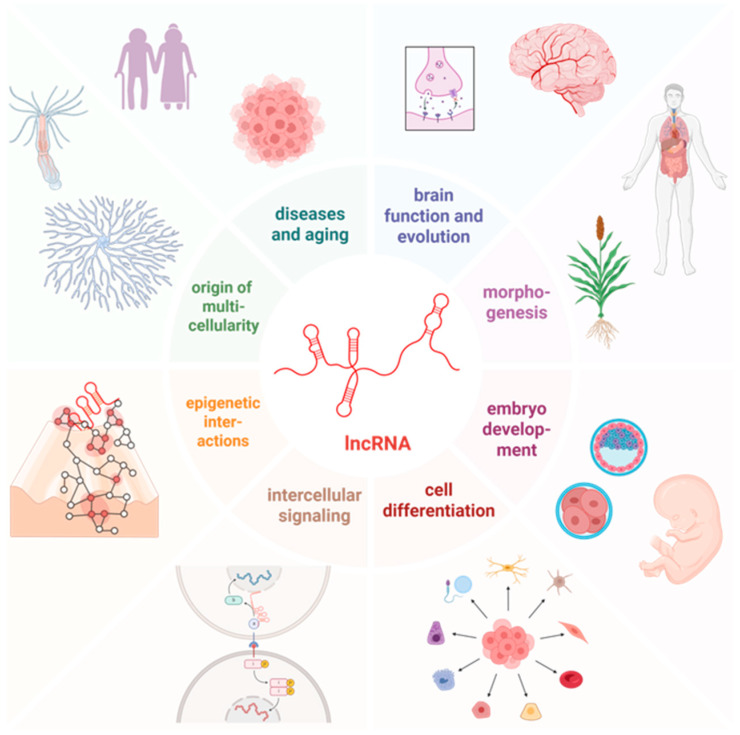
Conceptual synthesis of lncRNA-driven integration in multicellular systems. This diagram illustrates how disparate biological processes—from embryonic morphogenesis and cell-fate specification to cognitive evolution—are unified by a consistent RNA-centric integrative logic. By bridging molecular “fuzziness” (intrinsic disorder and conformational plasticity) with macroscopic tissue organization, lncRNAs act as the primary instructional scaffolds within the ISP framework. These networks underpin the precise spatial and temporal “rewiring” of the genome, maintaining organismal stability and homeostatic balance throughout the life cycle, from development to aging. Created in BioRender Zahmanova, G. (https://BioRender.com/wftne57, accesssed on 5 April 2026) is licensed under CC BY 4.0.

This RNA-centric integrative logic, which bridges molecular plasticity with the hierarchical complexity of multicellularity, is conceptually synthesized in Figure 2. Ultimately, lncRNAs provide the stable yet flexible control required for organismal stability. Their role as master architects of multicellularity reflects the integrative logic of genomic symbiosis observed at the subcellular level, now scaled to the dimensions of an entire organism.

### 5.2. IDPs, Phase Separation and Multicellularity

Within the ISP framework, lncRNAs are proposed to provide the instructional logic for complex life, while intrinsically disordered proteins (IDPs) serve as the biophysical “glue” that facilitates this integration. The observed correlation between the number of distinct cell types and the abundance of disordered residues suggests that the expansion of the “disordered proteome” was a fundamental prerequisite for organismal complexity [244].

Convergence through disorder: Even in the absence of sequence homology, proteins from lineages as phylogenetically distant as plants and animals frequently perform analogous roles by disorder-based functions [245,246]. For example, LEA proteins in plants and unique IDPs in tardigrades independently evolved the capacity to protect cellular integrity through vitrification [245,247]. This functional convergence through disorder is further exemplified by the independent evolution of analogous regulatory systems across kingdoms: from the disordered *KRP/p27^kip1^* inhibitors governing the cell cycle to the cryptochromes mediating circadian rhythms. In these systems, the transition from disorder to order appears to be a key regulatory strategy for managing complex biological information [245,248,249].

Organelle heterogeneity as a template for differentiation: The self-demixing of IDPs and RNAs creates specific “membraneless signatures” for different cell lineages. This heterogeneity is thought to act as a spatial template for cell-fate determinants, potentially driving developmental trajectories [250].

Systemic resilience “at the edge of chaos”: Multicellular systems utilize stress-inducible MLOs (e.g., stress granules) to synchronously “pause and restart” functions across diverse tissues—a mechanism fundamental to survival under environmental flux [251]. MLOs operate as open thermodynamic systems at the “edge of chaos.” Their metastability allows for near-instantaneous assembly, providing rapid, diffusion-based control of biochemical reactions [110,252,253]. However, this plasticity carries an inherent risk: failures in MLO homeostasis can lead to aberrant liquid-to-solid transitions, resulting in toxic aggregates (e.g., FUS, TDP-43) linked to neurodegeneration [110].

Causal emergence: Complexity emerges not by suppressing this disorder, but by organizing it into functional hierarchies [254]. As in Uversky’s model of causal emergence, multicellularity may transform micro-level stochasticity into macro-level stability [255]. This allows the organism to make reliable decisions—such as cell-fate determination—leveraging molecular “fuzziness”, rather than relying on rigid, specialized machinery.

In the following section, this multi-level symbiotic path is explored through concrete examples of integrated symbiotic pleiotropy (ISP)—molecular entities whose symbiotic origins underpin their sophisticated cellular functions.

## 6. The Symbiotic Path to Complexity: Case Studies

### 6.1. TERT: The Archetype of Multi-Level Symbiosis

Multi-level symbiosis and ISP relations: Telomerase reverse transcriptase (TERT), likely a “living fossil” from the ancient RNP world, represents an archetypal example of integrated symbiotic pleiotropy (ISP), as it functions at the intersection of molecular, genomic, organelle and cellular symbiosis.

- RNP nature and molecular domestication: Similar to Barbieri’s ribosoids, TERT operates as a ribonucleoprotein (RNP) complex where the lncRNA *TERC* provides the catalytic template [38,256]. This system is further regulated by TERRA, a telomeric lncRNA that acts as both a structural scaffold and a driver for the phase separation of telomeric chromatin [257,258]. Evolutionarily, the catalytic subunit of TERT originated from a “selfish” non-LTR retrotransposon, “domesticated” to resolve the end-replication problem of linear chromosomes [259].
- Mitochondrial “moonlighting”: In response to retrograde signaling under oxidative stress, TERT translocates to the mitochondria [260], where it protects the endosymbiont’s genome, aids in mtDNA replication, and modulates mtRNA transcription. Furthermore, TERT directly influences mitochondrial dynamics, affecting organelle shape, size, and fission/fusion dynamics [260,261].
- Systemic homeostasis: By maintaining telomere integrity and shielding the mitochondrial genome, TERT ensures the cellular vitality required for the existence of long-lived multicellular organisms. This coordination is central to tissue homeostasis: by balancing the trade-off between regenerative proliferation and programmed senescence, TERT-mediated condensates maintain the structural and functional integrity of the multicellular collective [262]. Its dysregulation in cancer highlights that the stability of TERT-containing “liquid hubs” is a fundamental requirement for organismal survival [263,264].

**Figure 3:**
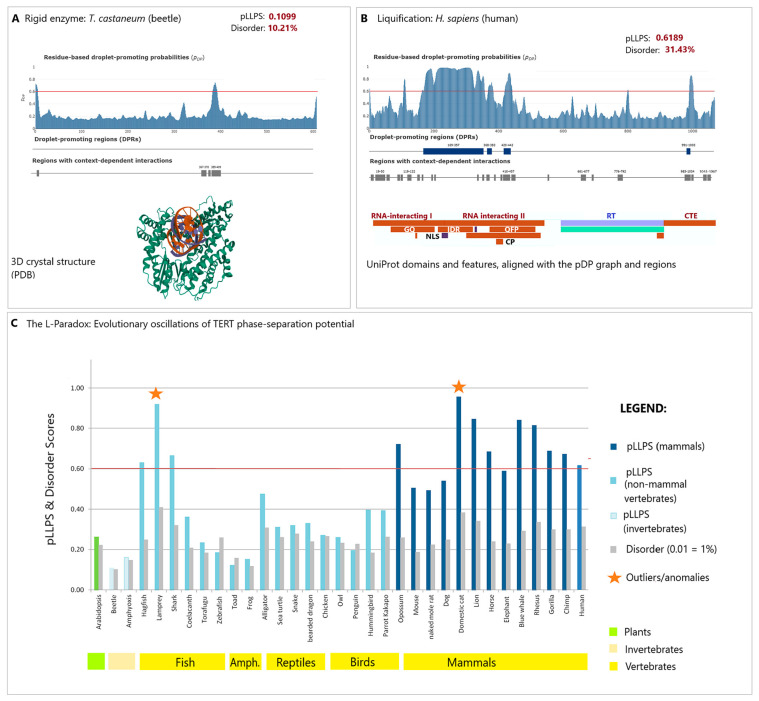
Evolutionary trajectory of TERT: from rigid catalyst to liquid-state driver. (**A**) Structural stability in invertebrates: The TERT ortholog from *T. castaneum* (PDB ID: 7QKM) represents the ancestral, predominantly ordered state. FuzDrop profiling reveals a low droplet-promoting probability (p_LLPS_: 0.11), where the protein acts as a rigid enzymatic unit. (**B**) LLPS expansion in mammals: Human TERT exhibits a significant rise in intrinsic disorder (31.43%) and p_LLPS_ (0.62). Domain mapping shows that liquidity peaks coincide with RNA-interacting motifs (GQ, CP, and QFP) and the Nuclear Localization Signal (NLS), suggesting that in higher eukaryotes, TERT functions as an active organizer of telomeric condensates. (**C**) The “L-paradox” across taxa: Large-scale analysis reveals that while intrinsic disorder remains relatively conserved (28–38% across vertebrates), the propensity for phase separation (pLLPS) exhibits sharp, lineage-specific oscillations. This “L-paradox” (liquidity paradox) indicates that phase behavior acts as an evolutionary “switch” or rheostat, with outliers like the lamprey and domestic cat (marked with red stars) showing extreme spikes in liquidity. This variability likely reflects lineage-specific adaptations in genomic maintenance, metabolic rate, and cellular longevity.

TERT phase separation patterns: Our expanded analysis of disorder and LLPS-related parameters across diverse taxa reveals a complex evolutionary trajectory that challenges linear models of biological complexity (Supplementary Table S2, Figure 3).

Rigid structure in invertebrates vs. “liquefication” in vertebrates: In invertebrates such as *T. castaneum*, TERT remains a predominantly ordered enzymatic unit (pLLPS: 0.11), functioning as a “passenger” within nuclear compartments (Figure 3A,C). In vertebrates, we observe a general trend toward “liquefication” (Figure 3B), though this progression is lineage-specific rather than linear (Figure 3C).

Oscillations in basal lineages: Basal lineages, including agnathans (lampreys) and cartilaginous fishes, exhibit unexpectedly high liquidity potential, where TERT crosses the critical droplet-promoting threshold (pLLPS > 0.6). Interestingly, this potential significantly decreases in bony fishes, amphibians, reptiles, and birds, only to “surge” again in mammals (Figure 3C).

Mammalian “fuzziness”: In humans, this biophysical “fuzziness” (31.43% disorder; pLLPS: 0.62) is concentrated within the RNA-interacting domains (GQ, CP, and QFP motifs) and the Nuclear Localization Signal (NLS) (Figure 3B). This suggests that in mammals, TERT has evolved from a mere client of TERRA-mediated phase separation into an active organizer and driver of telomeric condensates. Whether this increased liquidity is linked to the mitochondrial moonlighting of TERT remains a compelling subject for future investigation.

The L-Paradox: A mechanism for evolutionary switching? Our analysis reveals what we term the “L-paradox” (liquidity paradox): while intrinsic disorder remains relatively stable across vertebrate orthologs (typically 28–38%), the probability of spontaneous phase separation (p_LLPS_) exhibits dramatic, lineage-specific oscillations (Figure 3C). Despite relatively low sequence conservation (ranging from 60 to 80% in Eutheria to as low as 30% in non-mammalian vertebrates (data from Ensembl, Version 15.10, the stability of the disorder percentage suggests a sophisticated regulatory mechanism. We interpret these p_LLPS_ oscillations as an evolutionary switch or “rheostat” that recalibrates telomere maintenance to meet the specific longevity, metabolic rate, and regenerative demands of each clade.

### 6.2. RAG1: Exaptation and Phase Separation in the Immune System

Multi-level symbiosis and ISP relations. The recombination activating gene 1 (RAG1) is a key factor in biological complexity, marking a transition from level 2 (genomic symbiosis) toward the systemic requirements of level 4 (multicellularity). By enabling the generation of a vast repertoire of immune receptors, RAG1 serves as a foundational molecular engine for adaptive immunity. This capability is central to the stability of complex multicellular life, where the integrity of the collective relies on a sophisticated “self” vs. “non-self” recognition system to maintain homeostasis and provide defense against malignancy or external pathogens [265].

Double exaptation: RAG1 represents a remarkable case of both a “selfish” Transib transposase and its specific recognition sequences co-opted and synchronized approximately 500 million years ago. In this evolutionary partnership, the ancestral enzyme was domesticated into the RAG1 recombinase, while its terminal inverted repeats (TIRs) evolved into the modern recombination signal sequences (RSS) [266,267]. Within the ISP framework, this illustrates how a once parasitic genetic element is repurposed into a core biophysical component of vertebrate adaptive immunity.

RNP dynamics and recombination factories: RAG1 functions as a sophisticated ribonucleoprotein (RNP), where its RNA-binding capacity acts as a regulatory switch. It interacts with its partner RAG2 and with lncRNAs that scaffold “recombination factories,” preventing genomic instability by compartmentalizing DNA-cleavage activity through phase separation. [267,268].

RAG1 phase-separation patterns: Our analysis reveals that RAG1 operates at a critical biophysical threshold, with p_LLPS_ values (0.44–0.58 in most mammals) maintained just below the point of spontaneous condensation. This “borderline” state suggests that RAG1 bio-condensation is not random, but strictly context-dependent, requiring molecular “triggers” like lncRNAs to initiate recombination. Despite remarkably high sequence conservation across placental mammals (>90% identity) (data from Ensembl), our analysis suggests a sophisticated, non-linear optimization of RAG1 biophysics (Supplementary Figure S1, Supplementary Table S3):

- Threshold liquidity in higher primates: Humans and gorillas exhibit RAG1 profiles poised at the very edge of the liquid-state threshold (pLLPS 0.57–0.58). Notably, the gorilla achieves this high liquidity through a more “efficient” sequence, maintaining a lower overall disorder (32%) compared to the 34% in other primates (Table S3).
- The paradox of the Giants: The most striking divergence occurs between the world’s largest mammals, representing two contrasting evolutionary strategies: The blue whale (*Balaenoptera musculus*) stands out as the only species in our dataset where RAG1 crosses the high-confidence threshold (p_LLPS_ 0.6). In the vast cellular landscape of the blue whale, this “hyper-liquidity” may facilitate ultra-efficient immune surveillance. By lowering the energy barrier for condensate formation, the whale can rapidly shuffle its immune repertoire to suppress malignancy. In stark contrast, the Indian elephant (*Elephas maximus*) exhibits remarkably low RAG1 liquidity (p_LLPS_ 0.27). This suggests a “controlled fidelity” strategy. For the elephant, the priority is, most probably, the prevention of erroneous DNA cleavage. Most probably, by keeping RAG1 firmly in a non-spontaneous state, the elephant minimizes the risk of genomic instability, relying on slow but high-fidelity recombination to sustain its long-term survival.

Metabolic integration and organismal identity: RAG1 activity is further governed by mitochondrial health, acting as a metabolic sensor. Mitochondria regulate RAG1 stability via calcium-dependent phosphorylation, ensuring that DNA cleavage and recombination proceed only when metabolic fitness is guaranteed [269,270]. At the organismal scale, this “domesticated” machinery maintains the integrity of the multicellular collective by enabling the distinction between “self” and “non-self,” preventing internal destabilization by pathogens or malignancy [271].

In conclusion, the biophysical “tuning” of RAG1 offers a plausible molecular basis for the “quantal theory” of immunity, where systemic self/non-self discrimination is governed by discrete, all-or-none cellular decisions [265]. Within the ISP framework, we propose that the threshold-dependent phase separation of the RAG complex could act as the fundamental “quantal switch”, necessary for biological identity, potentially ensuring that the “quantal” threshold of immune recognition is calibrated to the specific metabolic and immunity requirements of the organism.

### 6.3. Arc: The Viral Capsid and the Synaptic Plasticity

The activity-regulated cytoskeleton-associated protein (Arc) represents the ultimate form of “biological networking.” By internalizing a viral-like capsid mechanism, Arc integrates ancient retroviral strategies into the complex cognitive architecture of animal brains.

Multi-level symbiosis and ISP relations. Arc represents a definitive integration of level 2 (genomic symbiosis) into the most complex architecture of multicellular organisms (level 4)—the cognitive brain. Originating from an ancient invasion by Ty3/gypsy retrotransposons, Arc was co-opted to regulate synaptic plasticity by functioning as a master scaffold bridging lncRNAs and the synaptic proteome. In synapses, Arc self-assembles into virus-like ribonucleoprotein (RNP) complexes that encapsulate transcripts required for synaptic remodeling [272,273,274]. This mechanism mirrors the gene transfer agents (GTAs) in prokaryotes—viral elements exapted by bacteria for horizontal information exchange [153,154]. Notably, this is a prime example of convergent evolution: while vertebrate Arc and *Drosophila* dArc1 originated from distinct retrotransposon lineages, both utilize capsid-like viral architecture for trans-synaptic RNA transport [274].

Endosymbiotic integration: The lifecycle of Arc capsids reveals a deep integration with the cell’s endosymbiotic history, representing a multi-genealogical crossroad: an ancient archaeal-derived system (ESCRT) processes a retrotransposon-derived capsid across cellular membranes, whose lipid landscapes were likely expanded and remodeled following the mitochondrial endosymbiosis [36]. This process, fueled by the enhanced eukaryote bioenergetics, effectively integrates three distinct symbiotic lineages to enable the high-order intercellular communication necessary for memory [275].

The vehicle of cognition: By delivering its RNA cargo across the synaptic cleft, Arc allows neurons to synchronize gene expression and coordinate long-term potentiation (LTP)—the cellular basis of memory consolidation. This domesticated viral mechanism acts as a physical vehicle for information transfer, enabling the stable storage and distribution of neural representations [276]. Within the ISP framework, Arc illustrates how the calibration of synaptic strength across neuron boundaries—and thus the very foundation of long-term memory and complex social behavior—would remain unattainable without the recruitment of these ancestral symbiotic legacies.

**Figure 4:**
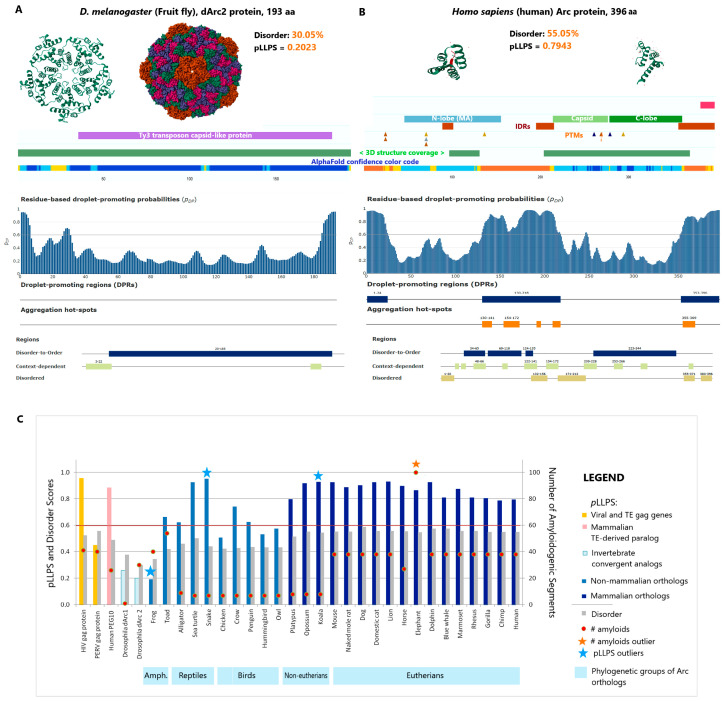
Biophysical landscapes of Arc orthologs and Gag-related proteins. (Top) 3D structures (PDB), residue-based droplet-promoting probabilities (p_DP_), predicted regions of disorder, aggregation, and context-dependent binding (FuzDrop/FuzPred), and UniProt domains/features aligned with the p_DP_ graph of (**A**) *D. melanogaster* (dArc2) and (**B**) *H. sapiens* (Arc), illustrating the evolutionary transition from rigid invertebrate architecture to high-disorder and LLPS propensity mammalian condensates. (Bottom) (**C**) Comparative analysis of p_LLPS_ propensity, protein disorder, and amyloidogenic potential across 30+ species. Left Y-axis: p_LLPS_ and disorder scores (0.0–1.0). Right Y-axis: Number of amyloidogenic segments (PASTA 2.0). Yellow/Pink bars: Evolutionary benchmarks (HIV-1 Gag, PERV Gag, and PEG10) revealing the high ancestral propensity for phase separation and aggregation. Light-blue bars: *Drosophila* convergent homologs (dArc1, dArc2); dark-blue bars: vertebrate orthologs, highlighting the significant “mammalian shift” in p_LLPS_. Red dots: Amyloidogenic potential, showing discrete “quantal” plateaus (7–8 for Sauropsids vs. 38 for Eutherians). Stars: Red and blue stars denote significant outliers in amyloidogenic potential (notably the Indian elephant) and p_LLPS_ propensity, respectively. Note: The proposed evolutionary trajectories and “quantum leaps” of these biophysical parameters are discussed in detail within the main text (Section 6.3).

Phase separation and aggregation landscapes: Beyond the evaluation of structural disorder (PONDR) and liquid–liquid phase separation (FuzDrop), we conducted a high-resolution analysis of the amyloidogenic potential of Arc and related gag-derived viral/transposable proteins using the PASTA 2.0 algorithm [277]. This additional layer of analysis is critical for Arc, as its unique function in the vertebrate brain—transitioning from dynamic signaling to stable synaptic tagging—probably relies on the controlled maturation of liquid condensates into more ordered, amyloid-like assemblies. Besides Arc, our analysis includes HIV-1 Gag (viral), PERV Gag (transposon-derived), and PEG10 (a domesticated mammal-specific retrotransposon) as evolutionary benchmarks. These controls allow us to map the transition from “aggressive” viral self-assembly (high p_LLPS_) to the regulated, context-dependent phase separation of the “domesticated” neuronal Arc (Figure 4, Supplementary Table S4).

Taxon-dependent clustering: Our results show clear phylogenetic clustering of p_LLPS_ values (Figure 4C). While invertebrate analogs (*Drosophila* dArc1/2, p_LLPS_ ≈ 0.20) rely on rigid, crystal-like oligomerization, vertebrate evolution marks a significant “liquefication” (Figure 4A,B).

The “mammalian shift”: A significant jump in p_LLPS_ potential occurs in mammals (almost all mammals showing p_LLPS_ values > 0.8). In turn, amyloidogenic potential shows clear “quantum” behavior: while birds, reptiles, monotremes and marsupials generally maintain lower amyloid counts (7–8), placental mammals stabilize at a “standard” of approx. 38 amyloidogenic segments, suggesting a baseline requirement for stable protein–protein interactions in complex brains (Figure 4C, Supplementary Table S4).

The reptile anomaly: Intriguingly, snakes and sea turtles exhibit “mammalian-like” high p_LLPS_ values (reaching ~1.0 in snakes). This suggests a convergent biophysical strategy, potentially optimized for rapid information processing in these lineages, despite their lower metabolic rates compared to mammals. In contrast, the sea turtle’s high p_LLPS_ regime (approaching 1.0) creates an interesting evolutionary counterpoint. While not typically associated with significant cognitive complexity, its extreme reliance on long-distance, innate geospatial navigation for nesting may necessitate unique, context-dependent synaptic stabilization. This disparity between snakes and turtles perfectly illustrates the L-paradox: a high potential for phase separation (p_LLPS_) is a molecular tool, not a single determinant of organismal complexity, whose functional outcome is broadly defined by the cellular and cognitive context.

Primate fine-tuning: In higher primates, we observe a stabilizing “fine-tuning,” with values shifting from the macaque (0.81) to a slightly lower, more controlled regime in humans (0.79). This subtle reduction likely prevents stochastic “noise,” ensuring that phase transitions occur only under strict synaptic signaling.

The elephant’s “hard drive”: The most prominent outlier in the amyloidogenic potential is the Indian elephant (*Elephas maximus*). Its Arc profile reveals a record-breaking amyloidogenic potential (100 segments). Even accounting for potential false positives in high-sensitivity settings, the magnitude of this peak is unique. This “biophysical stiffening” suggests that elephant memory may strongly rely on a “solid-state” archival strategy, where synaptic traces are rapidly converted into highly stable, amyloid-like structures to support their legendary long-term memory. Validation of these amyloidogenic peaks using PASTA 2.0, and the best energy scores further distinguishes the evolutionary strategies. While most vertebrates maintain a consensus best energy of −6.954, suggesting a regulated and potentially reversible amyloid-like state, the Indian elephant exhibits a significantly more stable profile (−7.818). This again points toward a highly stabilized, “long-term archival” state of the Arc-hub (Supplementary Table S4).

The mystery of the frogs: Interestingly, amphibians (*Xenopus* and *Bombina*) show the most extreme best energy values (−9.462) and significant amyloid segments (over 40), hinting at an ancestral reliance on ultra-stable, near-crystalline Arc assemblies during the early evolution of the vertebrate synapse (Supplementary Table S4).

In summary, Arc represents a remarkable case of molecular exaptation: evolution has repurposed this viral relic into a structural scaffold that stabilizes the physical traces of experience.

The evolution of TERT, RAG1, and Arc illustrates François Jacob’s principle of molecular tinkering—the idea that evolution functions by reshaping existing components and mechanisms into new roles [278,279]. We propose, however, that this process is a direct manifestation of a broader logic: integrated symbiotic pleiotropy. While tinkering describes *how* genetic materials and ancestral mechanisms are repurposed, our framework identifies the cause of this versatility in the symbiotic origins of the cell. In this view, these “parts” are not just passive fragments but formerly autonomous symbiotic legacies whose inherent multi-functionality drives the systemic integration of life’s complexity.

## 7. Discussion

### 7.1. The Isomorphisms of Multi-Level Complexity

The core thesis of this work is that eukaryotic complexity is not a mere accumulation of genetic “parts,” but a direct consequence of integrated symbiotic pleiotropy (ISP). This framework integrates four hierarchical levels of symbiosis—molecular, genomic, intracellular (endosymbiosis), and multicellular—linking disparate system components into a functional whole. These symbiotic levels span the entire biological landscape, yet they are most prominently expressed through lncRNAs, IDPs, and MLOs.

We propose a fundamental conceptual isomorphism across four levels of biological organization:

1. Informational level: Overlapping genetic codes and code degeneracy (Trifonov’s redundancy).
2. Molecular level: RNA and protein “moonlighting,” where single sequences perform multiple biological roles.
3. Interactive level: The multiconformational flexibility and multivalency of IDPs.
4. Systemic level: The fluidity of MLOs and the decentralized logic of RNP condensates.

At each level, pre-existing codes “leave space” for additional layers of information to be superimposed. Just as genetic degeneracy allows additional regulatory codes to be written onto the same nucleotide sequence [87], the multivalency of IDPs and MLOs provides the necessary “fuzziness” and degrees of freedom to integrate multiple physiological decisions. This multi-level overlapping is the biological equivalent of lossless data compression. By integrating maximum functional density into a single physical entity, the symbiotic cell overcomes the energetic and spatial constraints that limit prokaryotic simplicity. In this view, lncRNAs and IDPs are not “evolutionary noise” but the essential multi-dimensional software (the multi-level symbiotic toolkit) that integrates diverse symbiotic lineages—viral, archaeal, and bacterial—into a coherent physiological unit.

### 7.2. From Molecular Tinkering to Symbiotic Integration

While François Jacob’s concept of “molecular tinkering” [278,279] describes the trend of evolution repurposing existing materials, the ISP framework identifies the actual source of this versatility. The “parts” available for tinkering are not passive fragments; they are symbiotic legacies whose inherent multi-functionality is a direct consequence of their informational, functional, and systemic integration.

In our case studies—TERT, RAG1, and Arc—molecular domestication transcends the concept of tinkering and is more deeply understood as a core integrative process within the ISP framework. For instance, the transition from the “solid-state” rigid structures of invertebrates to the dynamic “liquid-state” mammalian hubs—as observed in our TERT and Arc analyses—represents a fundamental evolutionary shift toward increased symbiotic integration and regulatory complexity.

**Figure 5:**
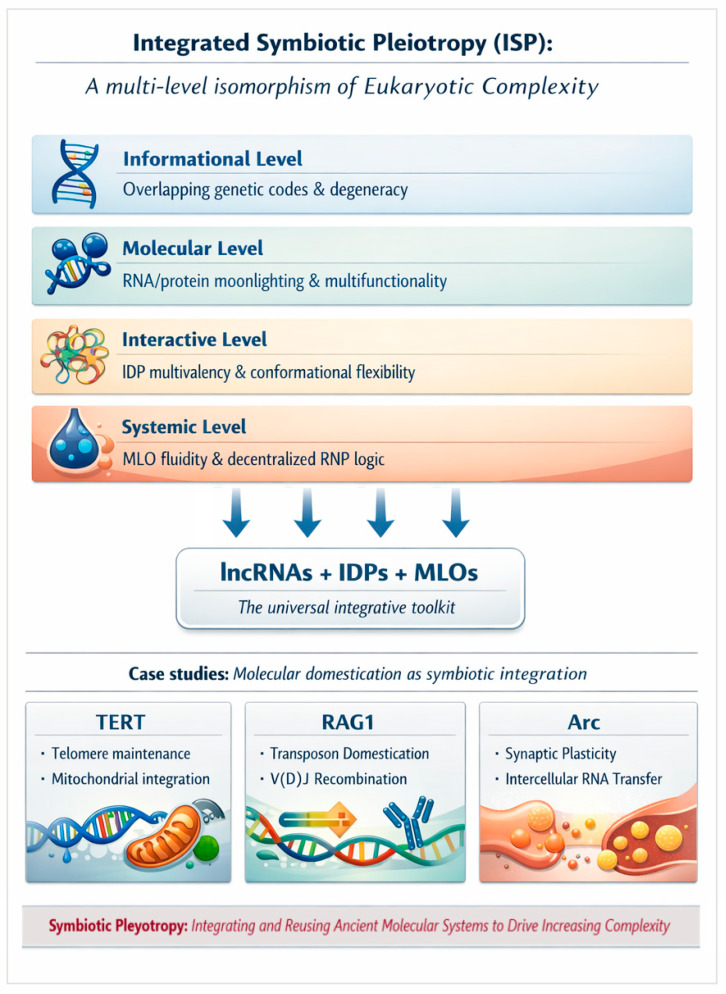
Visualization of the integrated symbiotic pleiotropy (ISP) framework. The four functional levels of isomorphism (informational to systemic) represent the conceptual integration of the four nested levels of symbiosis (molecular, genomic, intracellular, and intercellular) discussed throughout the manuscript and illustrated in Figure 1. Within the ISP framework, these levels are most prominently manifested through the universal integrative toolkit (lncRNA–IDP–MLO) of the modern eukaryotic cell. This multi-level isomorphism is further illustrated through hallmark examples of molecular domestication—TERT, RAG1, and Arc—ancient elements that have been repurposed to drive increasing biological complexity.

A visual synthesis of the main ideas within the ISP framework is represented in Figure 5.

### 7.3. Limitations of the ISP Model

To critically appraise the ISP framework, several inherent limitations must be addressed:

Publication and annotation bias: The perceived significance of lncRNAs, IDPs, and MLOs may be partially amplified by the intensive research focus on human and model organisms in these areas. Analytical prudence, coupled with conservative bioinformatic thresholds, is necessary to mitigate this “knowledge inflation.”

The predictive gap: Inferring precise biological function solely from disorder and pLLPS predictions remains challenging. While these tools identify the potential for phase separation, the actual in vivo state is heavily modulated by local stoichiometry, post-translational modifications, and other extrinsic cellular factors yet to be fully characterized.

Evolutionary causality: Assigning definitive causality from present-day molecular features is inherently speculative. However, by using evolutionary benchmarks (TERT, RAG1, Arc, and viral/transposon controls) and tracking the trends of disorder and LLPS in standard biophysical parameters across clades, we provide a robust comparative framework that moves beyond simple correlation toward a semiotic understanding of these ancient evolutionary trends.

### 7.4. Future Frontiers and Questions

Expanding to the holobiont: The principles of ISP appear to be scale-invariant. While this review focuses on eukaryotic multicellularity, the logic of symbiotic integration extends to the holobiont—the integrated ecosystem of the host and its microbiome, increasingly regarded in contemporary evolutionary theory as a significant unit of selection [280,281]. Here, metagenomic complexity represents a higher-order functional isomorphism, viewing the organism not as a discrete individual but as a macro-scale expression of the same integrative logic. The future incorporation of Symbiotic Level 5: The Holobiont into the ISP framework is intended to bridge the gap between molecular biology and ecosystem ecology, offering a path to close the evolutionary circle from the co-occurrence of genetic codes to the collective emergence of the biosphere.

Universal signatures of complexity: Beyond TERT, RAG1, and Arc, do the observed trends in p_LLPS_ profiles and the “L-paradox” represent universal signatures for other proteins driving complex multicellularity?

Systems medicine and pleiotropic pharmacology:

- Better understanding and modeling protein aggregation: What are the evolutionary trade-offs between intrinsic disorder, LLPS propensity and amyloidogenic potential required to prevent pathological transitions?
- Genome variation and LLPS: How do specific mutations alter the multi-level behavior of LLPS-engaging proteins in the contexts of evolution and personalized medicine?
- Symbiotic/pleiotropic drugs? Building upon the emerging concept of condensate modulators (c-mods) [282], future therapeutic strategies may transcend single-target interventions in favor of pleiotropic agents designed to modulate the overlapping functional codes of their targets. Alternatively, such therapies could target the collective biophysical state of symbiotic ensembles, thereby restoring the homeostatic balance of cells and tissues. However, to avoid systemic toxicity, the clinical success of these pleiotropic strategies will depend on context-dependent specificity—ensuring that such agents modulate biophysical ensembles only within defined pathological windows, rather than disrupting the multi-level symbiotic landscape of the organism.

## 8. Conclusions

Ultimately, we are not mere mechanistic hosts to ancient genetic fragments—we are the living manifestation of their successful cooperation. Understanding life through the lens of integrated symbiotic pleiotropy suggests that biological complexity is born from continuous, multi-level collaboration. In this light, the creativity of life is the remarkable ability to upgrade conflict into cooperation and to reshape evolutionary constraints into new horizons for innovation.

## 9. Materials and Methods

### 9.1. Sequence Retrieval and Ortholog Selection

- Sequence retrieval and ortholog validation: Protein sequences for TERT, RAG1, and Arc were retrieved from the NCBI RefSeq database [121]. One-to-one orthology was inspected via the NCBI RefSeq Orthologs interface [283]. For a representative subset, orthology was further validated through Ensembl Compara release 115 [284], checking for 1:1 reciprocal best hits and percentage identity to ensure consistent evolutionary mapping.
- Isoform selection: We prioritized MANE Select transcripts for human sequences. For other species, the longest protein-coding isoform was selected. For disorder predictions of MLO marker proteins, the longest isoform was selected, which in most cases is also the MANE Select isoform (Supplementary File, Sheet “MLO marker proteins”). For all TERT orthologs, where multiple different isoforms exist, we specifically selected the isoform with the highest predicted p_LLPS_ to explore the maximal symbiotic potential of the locus.

### 9.2. Intrinsic Disorder Prediction (PONDR)

Intrinsic disorder was calculated using the PONDR^®^ VL-XT algorithm [120]. A default threshold of 0.5 was applied; residues scoring ≥0.5 were classified as disordered. This threshold is the standard biophysical benchmark for binary classification, providing a balanced sensitivity for identifying Short Linear Motifs (SLiMs) within disordered regions.

### 9.3. Phase Separation Landscapes, Binding and Aggregation Propensity

Biophysical predictions for phase separation and interaction versatility were performed using the FuzDrop and FuzPred web servers [285].

Phase separation (FuzDrop): The propensity for liquid–liquid phase separation (LLPS) was quantified using default parameters [285].

- p_LLPS_: Represents the overall probability of a protein to act as a “droplet driver.” A threshold of ≥0.60 was applied, as this cutoff has been statistically optimized to maximize the separation between experimental LLPS drivers and non-segregating proteins, effectively minimizing false positives [285].
- p_DP_ (droplet-promoting probability): A residue-specific score used to map the internal “landscapes” of the protein. This identifies disordered segments that specifically drive the condensation process. The pDP profiles for TERT and Arc (Figure 3 and Figure 4) were extracted from the FuzDrop graphical output, with profiles for all TERT, RAG1 and Arc orthologs provided in Supplementary Tables S2–S4.
- Binding modes (FuzPred): Interaction versatility was assessed via FuzPred [286] (default settings), focusing on two key metrics:
- p_DO_ (disorder to order): The probability (≥0.60) of a disordered region undergoing a conformational transition (folding) upon binding [286].
- MBM (multiplicity of binding modes): A Shannon entropy-based metric (≥0.65) used to identify “fuzzy” regions. These segments are capable of context-dependent, multimodal interactions, reflecting high functional pleiotropy [286].
- Aggregation hot spots: In addition to droplet-promoting propensity, we identified aggregation hot spots [287] (orange blocks in Figure 4 and Supplementary Table S4) using the FuzDrop server. These regions represent sequence-specific motifs with a high thermodynamic propensity for spontaneous self-association. Unlike the broader disordered regions (DPRs) that drive liquid–liquid phase separation, these hot spots indicate a transition toward more structured, solid-like or amyloid-like assemblies.

### 9.4. Amyloidogenic Potential (PASTA 2.0)

Potential amyloid-forming segments for Arc, its ortholog, and gag-related proteins were predicted using PASTA 2.0 [288].

Settings: For “#amyloids”, “Top pairing energies” were set to 100 to achieve high-resolution differentiation between closely related mammalian clades. The best energy (PE) value was used to compare the thermodynamic stability of the amyloid cores across lineages. The default energy threshold of −5.0 was used to filter significant hits.

### 9.5. Protein 3D Structures and Domain Visualization

3D visualizations: Representative 3D structural visualizations for *T. castaneum* TERT (PDB ID: 7QKM), *Drosophila* dArc2 capsid-like structure (6TAQ), and human Arc segments (6TQ0, 6TN7) were obtained from the RCSB Protein Data Bank (PDB) database [289] using the integrated NGL viewer for molecular rendering.

Domain mapping: Functional domains and PTMs were identified via the UniProt Feature Viewer (ProtVista) version 4.7.0 [290]. As shown in Figures 3A,B and 4A,B, these maps were manually aligned with p_DP_ (FuzDrop) profiles to visually correlate biophysical properties with known structural motifs.

AlphaFold2 visualization: For the Arc case study, images of AlphaFold2-predicted Computed Structure Models (CSMs) [291] were used specifically as an alternative way to visualize levels of intrinsic disorder for fly and human Arc genes. Low-confidence regions (orange/yellow) were used as independent structural evidence to validate the high intrinsic disorder and “liquid” nature of the human Arc ortholog compared to the rigid, high-confidence (blue) “solid-state” structure of *Drosophila* dArc2 (Figure 4A,B).

## Figures and Tables

**Table 1:** Representative MLOs across eukaryotic cellular compartments and convergent structures in prokaryotes. The percentage of intrinsically disordered residues in the amino acid sequences of marker proteins was calculated using the PONDR^®^ software [120] for the longest protein isoform in the NCBI RefSeq Protein database [121] (Supplementary Table S1). Table 1 has been updated with the refined disorder scores based on the most recent protein-coding sequence annotations to provide a synchronized evolutionary comparison.“?” It is not known.

MLO	Localization, Kingdom	Function	Nucleic Acids	Marker Protein(s) (PONDR-Predicted Disorder %)	Reference
Nucleolus	Nucleus, Eukarya	rRNA transcription, processing, ribosome biogenesis	rRNA, PAPAS, SLERT	Nucleolin (55.5%), Fibrillarin (44.2%)	[122,123,124]
Nuclear (splicing) speckle	Nucleus, Eukarya	Pre-mRNA processing, gene expression regulation	MALAT1, 7SKRNA	SRSF1 (47.2%)	[122,123,124]
Paraspeckle	Nucleus, Eukarya	Chromatin and gene expression regulation, stress response	NEAT1, Linc-RNA-p21	NONO (63.3%), SFPQ (72.7%)	[124]
Nuclear stress body	Nucleus, Eukarya	Chromatin architecture alteration under stress	HSATIII	HSF1 (51.2%)	[24,25,26,122,123,124,125]
Histone locus body	Nucleus, Eukarya	Processing histone pre-mRNAs	Y3/Y3 RNA	NPAT (56.8%)	[24,25,26,122,123,124,125]
Cajal body	Nucleus, Eukarya	snRNP biogenesis, spliceosome assembly	TERC RNA, small CB-associated RNAs	Coilin (56.6%)	[122,123]
P body	Cytoplasm, Eukarya	mRNA storage, degradation, quality control, miRNA suppression	mRNAs	EDC3 (35.6%), DCP2 (25.6%)	[26]
Stress granule	Cytoplasm, Eukarya	Untranslated mRNA storage, protection, stress response	NORAD, mRNAs	eIF3A (66.4%), G3BP (53.2%)	[24,25,26,126]
Nucleoid	Mitochondrion, Eukarya	Recruitment of transcription machinery via co-phase separation	mtDNA	TFAM (40.7%)	[127]
MitoRNA granules (MRGs)	Mitochondrion, Eukarya	Mitochondrial ribosome biogenesis	dsRNA from mtDNA	GRSF1 (39.8%), FASTK (45.7%)	[127]
STT1/2-driven phase-separated compartment	Chloroplast, Eukarya (plants)	Sorting chloroplast proteins to thylakoid membranes	?	SECA1 (31.6%)	[128]
Pole organizer	Bacteria (*Caulobacter vibrioides*)	Scaffold, locks signal proteins to cell poles	?	popZ (89.3%)	[117,118,119]
FtsZ-SlmA-SBS droplets	Bacteria (*E. coli*)	Metabolite regulation, heat stress response	?	FtsZ (33.7%)	[118,119]
Dps condensate	Bacteria (*E. coli*)	Starvation stress response, dense DNA packing	DNA	Dps (29.9%)	[117,119]
Droplet-like DNA-protein condensate	Archaea (*Sulfolobus islandicus*)	Archaeal chromosome organization	DNA	Archaeal DNA condensing protein 1 (aDCP1) (in vitro)	[129]

## Data Availability

No new data were created or analyzed in this study. Data sharing is not applicable to this article.

## Supplementary Materials

The following supporting information can be downloaded at https://www.mdpi.com/article/10.3390/ijms27083478/s1.
