# Supplementary material for: Integrated Symbiotic Pleiotropy: Long Non-Coding RNAs and Disordered Proteins Interweaving the Functional Layers of the Eukaryotic Cell
Source: Int J Mol Sci. 2026 Apr 13;27(8):3478. doi: 10.3390/ijms27083478 (PMC13115813; doi:10.3390/ijms27083478)
Supplement: Supplementary file 1 [file ijms-27-03478-s001.zip › ijms-4219786-supplementary.pdf]

Supplementary Material

Integrated Symbiotic Pleiotropy: Long Non-Coding RNAs and Disordered Proteins Interweaving the Functional Layers of the Eukaryotic Cell

Evelina Daskalova[1], Joon Seon Lee[2], Gergana Zahmanov[1,3], and Ivan Minkov[3,4]

1 Department of Molecular Biology, University of Plovdiv, Plovdiv, 4000, Bulgaria; eve_das@uni-plovdiv.bg

2 Faculty of Sciences, Brigham Young University–Hawaii, Laie, HI 96762, USA; Joon.Lee@byuh.edu

3 Center of Plant Systems Biology and Biotechnology, Plovdiv, 4000, Bulgaria; gerganaz@uni-plovdiv.bg

4 Institute of Molecular Biology and Biotechnologies, Markovo, 4108, Bulgaria; minkov@plantgene.eu

Correspondence: minkov@plantgene.eu (I.M.); gerganaz@uni-plovdiv.bg (G.Z.)

Contents of this file:

- **Supplementary Table S1.** Localization, isoform data, and Disorder scores of key MLO-associated proteins across Eukarya, Bacteria, and Archaea.
- **Supplementary Table S2.** Droplet-promoting probability, Disorder scores and phase separation propensity for TERT orthologs and isoforms.
- **Supplementary Table S3.** Droplet-promoting probability, Disorder scores and phase separation propensity for RAG1 orthologs.
- **Figure S1.** Predicted p_{LLPS} and disorder scores among RAG1 orthologs.
- **Supplementary Table S4.** Droplet-promoting probability, Disorder scores, phase separation propensity and aggregation potential of Arc orthologs and related retrotransposon-derived sequences.

Supplementary Table S1. Localization, Isoform data, and Disorder scores of key MLO-associated proteins across Eukarya, Bacteria, and Archaea.

Cellular Localization / Taxon	MLO */Condensate Type	Protein Hame	No. of Protein Isoforms (NCBI)	No. of ncRNA Isoforms	Longest Isoform Length (aa)	RefSeq ID (Longest Isoform)	MANE Select Status	Disorder Content (%) [PONDR®]
Nucleus, Eukarya	Nucleolus	Nucleolin	1	0	710	NP_005372.2	Y	55.49
	Nucleolus	Fibrillarin	7	0	1125	NP_001427.2	Y	44.24
	Nuclear (splicing) speckle	SRSF1	10	4	321	NP_008855.1	Y	47.18
	Paraspeckle	NONO	4	0	471	NP_031389.3	Y	63.27
	Paraspeckle	SFPQ	11	2	707	NP_005057.1	Y	72.70
	Nuclear stress body	HSF1	26	0	557	XP_005272372.1	N	52.06
	Histone locus body	NPAT	4	0	1436	XP_011541156.1	N	56.76
	Cajal body	Coilin	1	0	576	NP_004636.1	Y	56.60
Cytoplasm, Eukarya	P body	EDC3	13	0	508	NP_079359.2	Y	35.63
	P body	DCP2	8	1	420	NP_689837.2	Y	25.56
	Stress granule	eIF3A	1	0	1382	NP_003741.1	Y	66.35
	Stress granule	G3BP	2	0	466	NP_005745.1	Y	53.22
Mitochondrion, Eukarya	Nucleoid	TFAM	6	1	246	NP_003192.1	Y	40.65
	MitoRNA granules (MRGs)	GRSF1	6	0 **	480	NP_002083.4	Y	39.79
	MitoRNA granules (MRGs)	FASTK	19	0	549	NP_006703.1	Y	45.72
Chloroplast, Eukarya (Plants)	STT1/2-driven phase-separated compartment	SECA1 (AGY1)	3	0	1042	NP_001190653.1	N	31.57
Bacteria (Caulobacter vibrioides)	Pole organizer	popZ			177	YP_002516753.1	-	89.27
Bacteria (E. coli)	FtsZ-SlmA-SBS droplets	FtsZ			383	NP_414637.1	-	33.68
Bacteria (E. coli)	Dps condensate	Dps			167	NP_415333.1	-	29.94
Archaea (Sulfolobus islandicus)	Archaeal chromosome organization	Archaeal DNA condensing protein 1 (aDCP1)			N/A	N/A	N/A	N/A

* **Abbreviations: MLO**, membraneless organelle; **NCBI**, National Center for Biotechnology Information; **MANE**, Matched Annotation from NCBI and EMBL-EBI; **PONDR**, Predictor of Natural Disordered Regions. **Y**, Yes; **N**, N/A **-** Not applicable.;

** One antisense (AS) transcript identified

Supplementary Table S2. Droplet-promoting probability, Disorder scores and Phase separation propensity for **TERT** orthologs and isoforms.

p_{DP} profiles and DPRs of TERT orthologs	Organism (No isoforms) Accession No	Protein sequence length, aa	Overal Disorder	p_{LLPS} score (FuzDrop) range*	Relation to human TERT
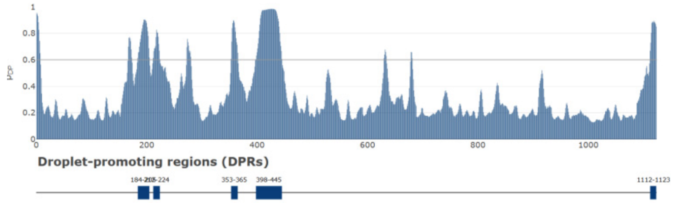	*Arabidopsis thaliana* (1) NP_197187	1123	22.35%	0.2631	Distant ortholog
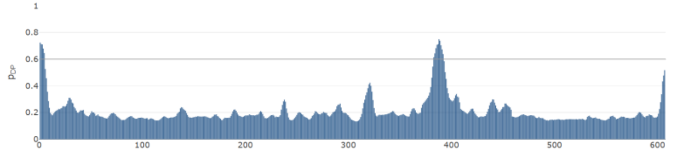	Red flour beetle *Tribolium castaneum* (1) XP_008199630.1	607	10.21%	0.1099	Distant ortholog
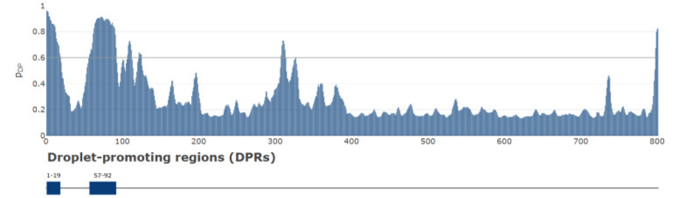	Amphyoxis *Branchiostoma floridae* (1) XP_035699554.1	801	14.86%	0.1620	Distant homolog
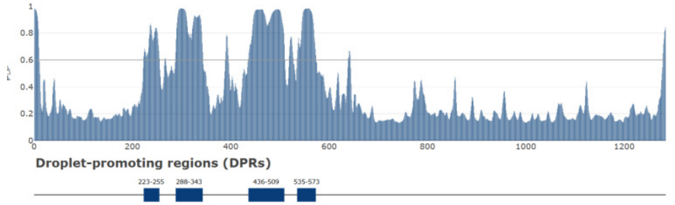	Atlantic hagfish *Myxine glutinosa* (2) XP_067983998.2	1289	24.90%	**0.6319**<br><br>range: 0.6260–0.6319	1:1 ortholog
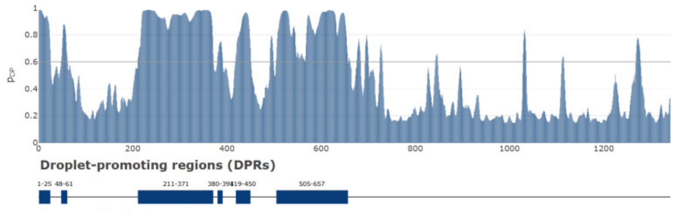	Lamprey *Petromyzon marinus* (1) XP_032822741.2	1341	41.01%	**0.9205 (!)**	1:1 ortholog
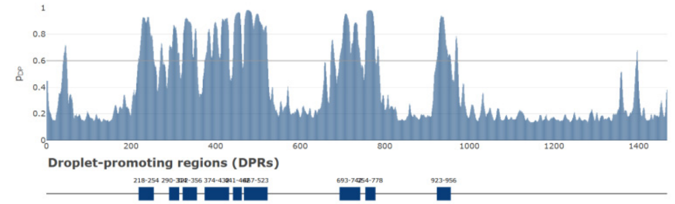	Great white shark *Carcharodon carcharias* (1) XP_041039955	1467	32.17%	0.6672	1:1 ortholog

p_{DP} profiles and DPRs of TERT orthologs	Organism (No isoforms) Accession No	Protein sequence length, aa	Overal Disorder	p_{LLPS} score (FuzDrop) range*	Relation to human TERT
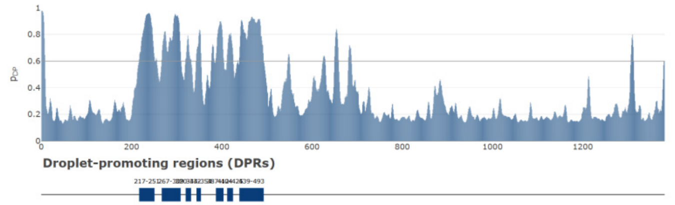	Coelacanth *Latimeria chalumnae* (1) XP_064410215.1	1381	20.93%	0.3632	1:1 ortholog
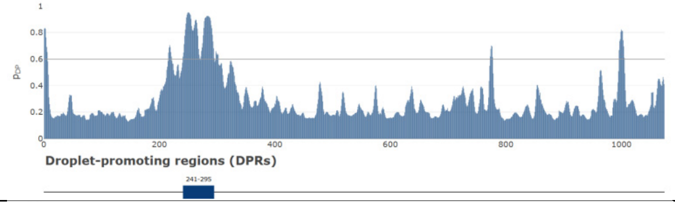	Torafugu fish *Takifugu rubripes* (3) XP_003969013.1	1074	18.53%	0.2350<br><br>pLLPS range: 0.1760–0.2350	1:1 ortholog
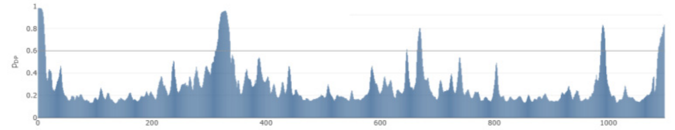	Zebrafish *Danio rerio*(1) NP_001077335.1	1098	25.96%	0.1860	1:1 ortholog
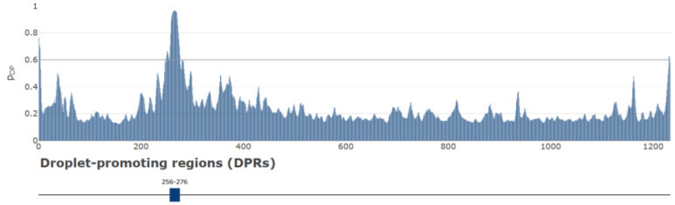	Fire-bellied toad *Bombina bombina* (1) XP_053571738.1	1233	15,90%	0.1234	1:1 ortholog
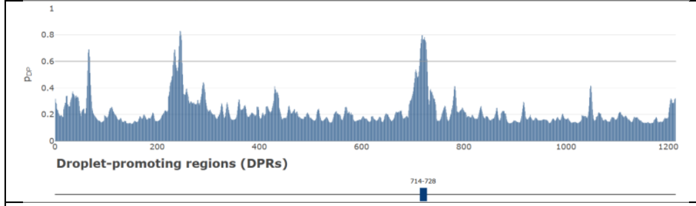	Tropical clawed frog *Xenopus tropicalis* (5) XP_017950465.1	1214	11.78%	0.1528<br><br>pLLPS range: 0.1445–0.1528	1:1 ortholog
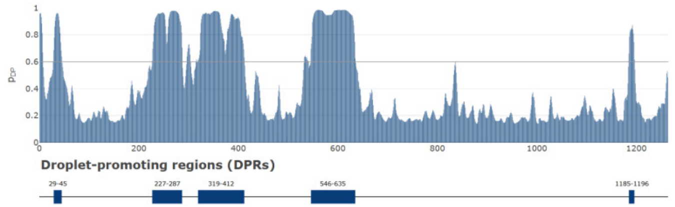	American alligator *Alligator mississippiensis* (3) XP_019346635.1	1263	30.96%	0.4764<br><br>range: 0.4426–0.4764	1:1 ortholog

p_{DP} profiles and DPRs of TERT orthologs	Organism (No isoforms) Accession No	Protein sequence length, aa	Overal Disorder	p_{LLPS} score (FuzDrop) range*	Relation to human TERT
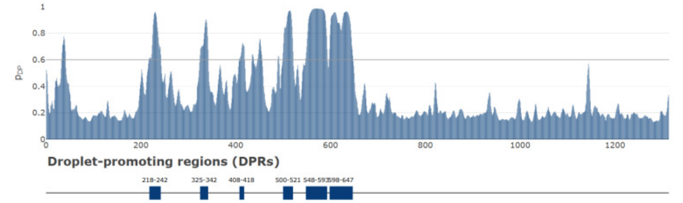	Central bearded dragon *Pogona vitticeps* (2) XP_072854562.2	1313	23.99%	0.3310<br><br>range:<br>0.3252–0.3310	1:1 ortholog
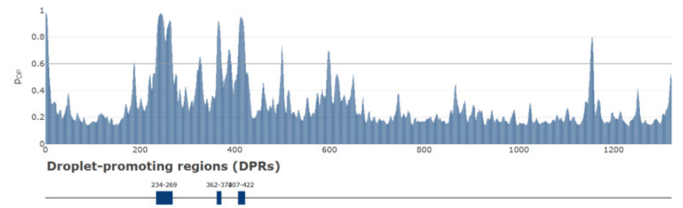	Green sea turtle *Chelonia mydas* (9) XP_043396234.1	1322	26.10%	0.3115<br><br>range:<br>0.2558–0.3115	1:1 ortholog
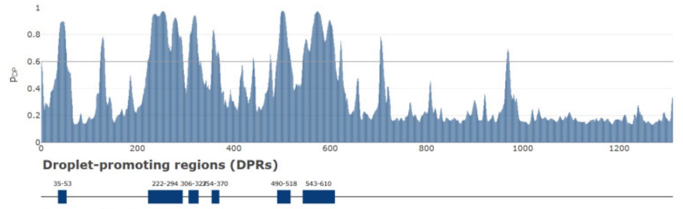	Snake (adder) *Vipera berus* (1) XP_081182233.1	1311	27.92%	0.3205	1:1 ortholog
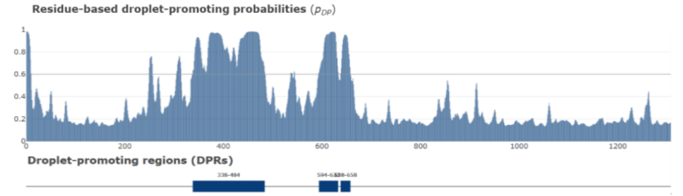	Chicken *Gallus gallus* **(12)** XP_040540215.1	1334	26.61%	0.2719<br><br>range:<br>0.1142–0.2719	1:1 ortholog
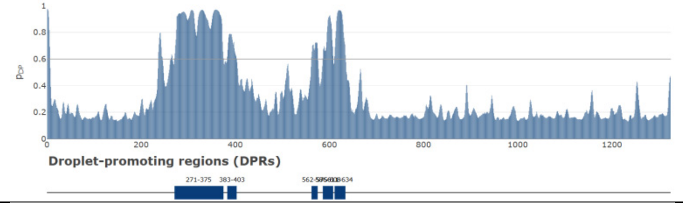	Tawny owl *Strix aluco* (1) XP_074680510.1	1324	23.41%	0.2609	1:1 ortholog
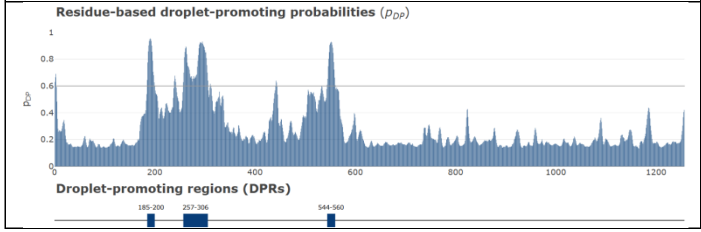	Emperor penguin *Aptenodytes forsteri* (1) XP_009278490.1	1256	22.85%	0.1968	1:1 ortholog

p_{DP} profiles and DPRs of TERT orthologs	Organism (No isoforms) Accession No	Protein sequence length, aa	Overal Disorder	p_{LLPS} score (FuzDrop) range*	Relation to human TERT
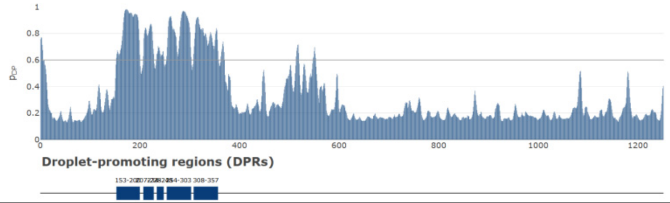	Parrot Kakapo<br>*Strigops habroptila* (2)<br>XP_030368452.1	1251	26.38%	0.3940<br><br>range:<br>0.3313–0.3940	1:1 ortholog
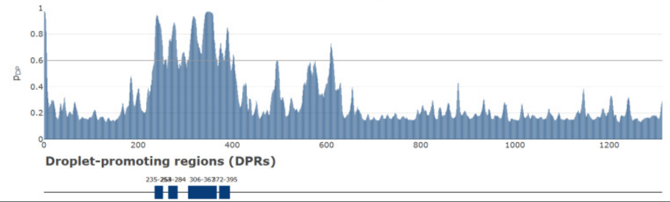	Anna's Hummingbird<br>*Calypte anna* (1)<br>XP_030300906.1	1312	18.52%	0.3974	1:1 ortholog
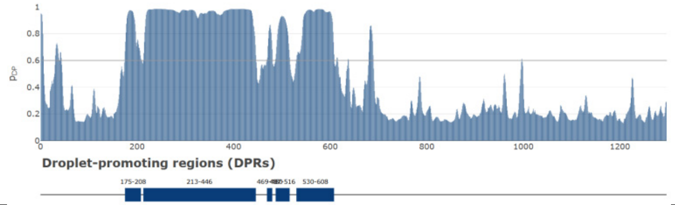	Platypus<br>*Ornithorhynchus anatinus* (1)<br>NP_001269394.1	1296	37.27%	0.6334	1:1 ortholog
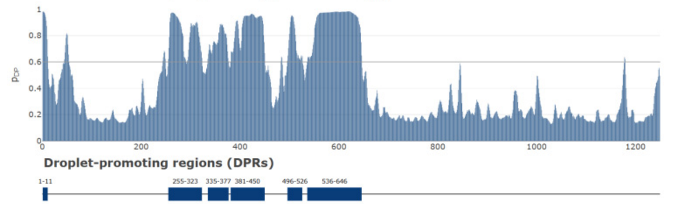	Opossum<br>*Monodelphis domestica* (3)<br>XP_056680302.1	1249	26.02%	0.7236<br><br>range:<br>0.7030–0.7236	1:1 ortholog
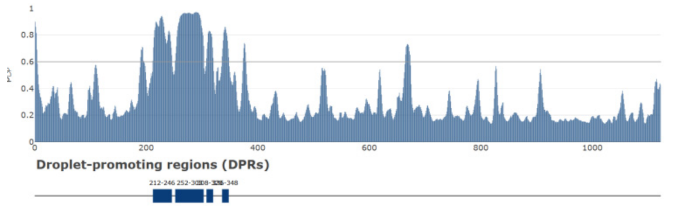	House mouse<br>*Mus musculus* (3)<br>NP_033380.1	1122	18.81%	0.5054<br><br>range:<br>0.1610–0.5054	1:1 ortholog
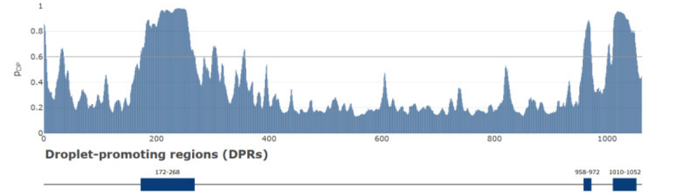	Naked mole rat<br>*Heterocephalus glaber* (3)<br>XP_021114238.1	1061	22.43%	0.4949<br><br>range:<br>0.3227–0.4909	1:1 ortholog

p_{DP} profiles and DPRs of TERT orthologs	Organism (No isoforms) Accession No	Protein sequence length, aa	Overal Disorder	p_{LLPS} score (FuzDrop) range*	Relation to human TERT
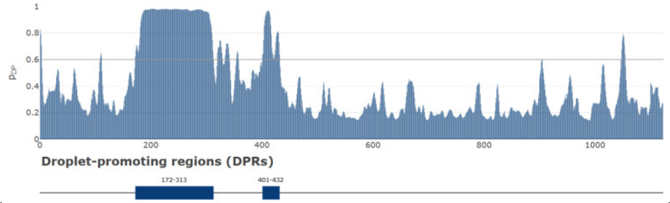	Domestic dog<br>*Canis lupus familiaris* (1)<br>NP_001026800.2	1122	24.96%	0.5409	1:1 ortholog
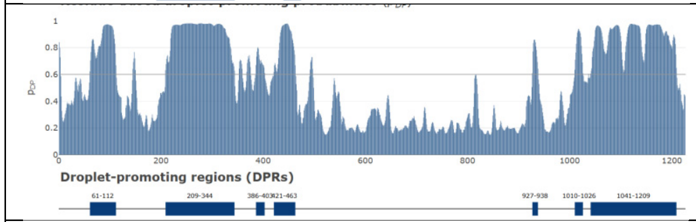	Domestic cat<br>*Felis catus* (15!)<br>XP_023095585.2	1126	38.34%	**0.9569 (!!!)**<br><br>range: 0.7750–0.9569	1:1 ortholog
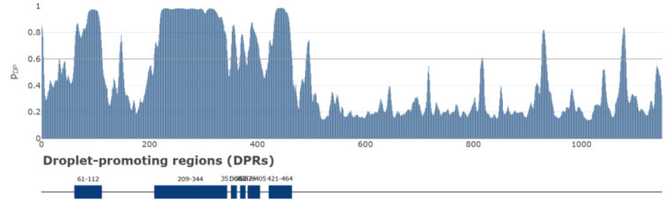	Lion<br>*Pantera leo* (1)<br>XP_042759391.1	1149	34.12%	**0.8466**	1:1 ortholog
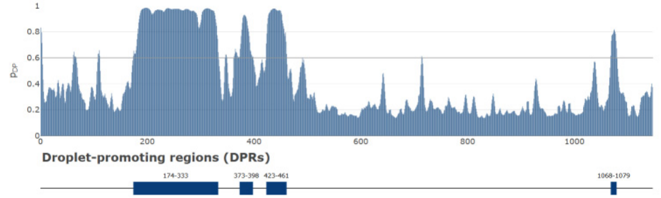	Horse<br>*Equus caballus* (2)<br>XP_023481651.1	1122	24.06%	0.6869<br><br>range:<br>0.6166–0.6869	1:1 ortholog
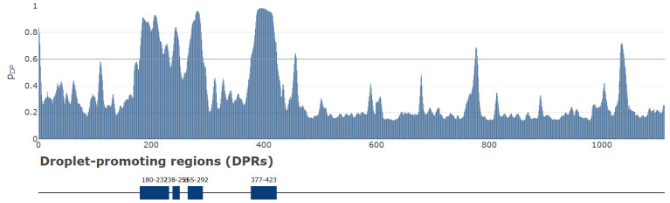	Indian elephant<br>*Elephas maximus* (1)<br>XP_049717117.1	1111	22.95%	0.5903	1:1 ortholog
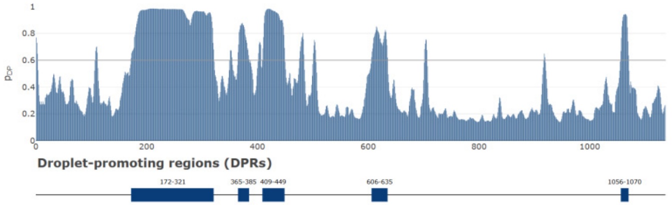	Blue whale<br>*Balaenoptera musculus* (1)<br>XP_036701283.1	1136	29.31%	**0.8425**	1:1 ortholog

p_{DP} profiles and DPRs of TERT orthologs	Organism<br>(No isoforms)<br>Accession No	Protein sequence length, aa	Overal Disorder	p_{LLPS} score (FuzDrop) range*	Relation to human TERT
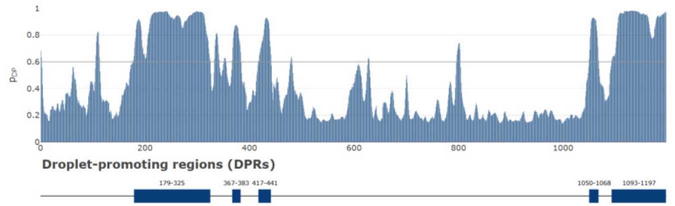	Rhesus monkey<br>*Macaca mulatta* (3)<br>XP_077860306.1	1197	33.67%	**0.8161**<br><br>range:<br>0.6514–0.8161	1:1 ortholog
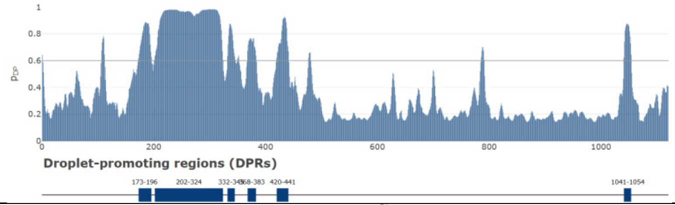	Western lowland gorilla<br>*Gorilla gorilla gorilla* (2)<br>XP_055222862.1	1120	30.00%	0.6890<br>range:<br>0.6087–0.6890	1:1 ortholog
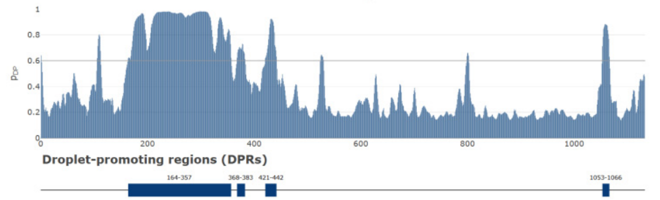	Chimp<br>*Pan troglodytes*<br>XP_016808391.2	1132	29.95%	0.6738	1:1 ortholog
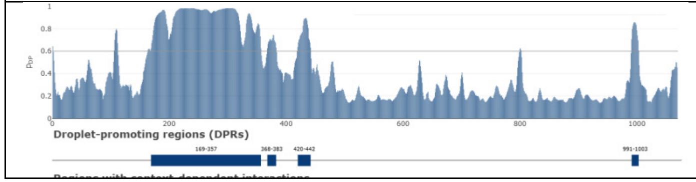	Human<br>*Homo sapiens* (2)<br>NP_001180305.1	1069	31.43%	0.6189<br><br>range:<br>0.5917–0.6189	

⁎ **range:** The values represent the minimum and maximum p_{LLPS} scores observed among all analyzed protein isoforms. The primary score highlighted in the table corresponds to the isoform with the highest predicted propensity for phase separation.

Note: For further discussion of these regions, see Main Text (Section 6.1).

Supplementary Table S3. Droplet-promoting probability, Disorder scores and Phase separation propensity for **RAG1** orthologs.

**p_{DP} profiles and DPRs of RAG1 orthologs**	**Organism**<br>**Accession No**	**Sequence length, aa**	**Overal Disorder**	**p_{LLPS} score**	**Relation to human RAG1**
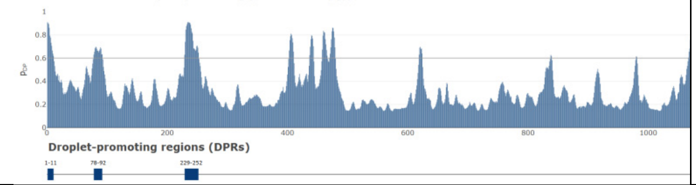	Great white shark<br>*Carcharodon carcharias*<br>XP_041054125	1072	34.24%	0.2047	1:1 ortholog
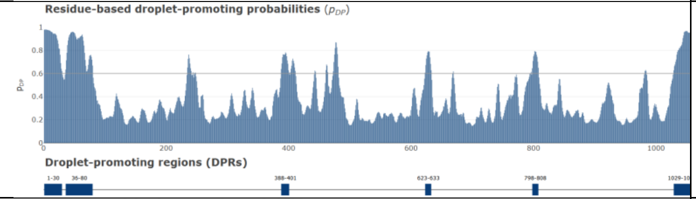	Zebrafish<br>*Danio rerio*<br>NP_571464	1057	39.64%	0.4876	1:1 ortholog
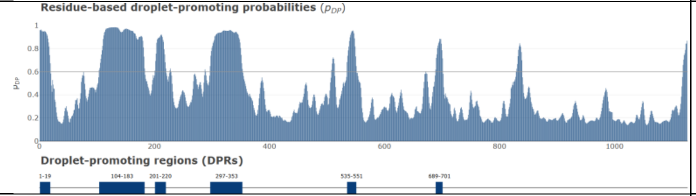	Tropical clawed frog<br>*Xenopus tropicalis*<br>XP_002937338	1126	36.06%	0.5656	1:1 ortholog
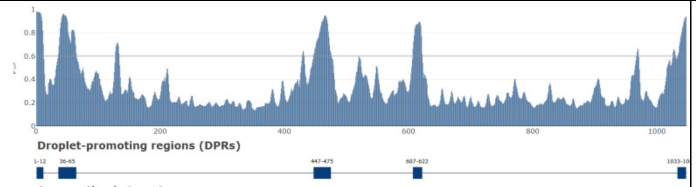	Green sea turtle<br>*Chelonia mydas*<br>XP_007055298	1047	32.28	0.2764	1:1 ortholog
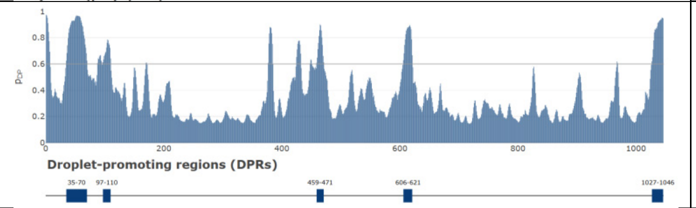	Snake (adder)<br>*Vipera berus* (1)<br>XP_081183968.1	1046	33.27%	0.2588	1:1 ortholog
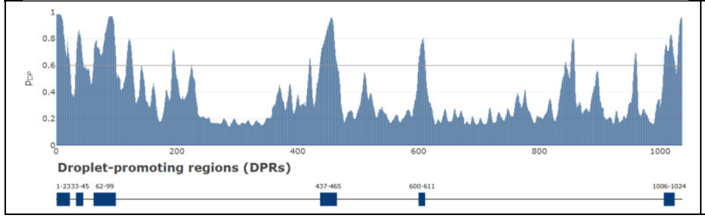	Emperor penguin<br>*Aptenodytes forsteri*<br>XP_009280732.1	1036	31.37%	0.3509	1:1 ortholog

**p_{DP} profiles and DPRs of RAG1 orthologs**	**Organism Accession No**	**Sequence length, aa**	**Overal Disorder**	**p_{LLPS} score**	**Relation to human RAG1**
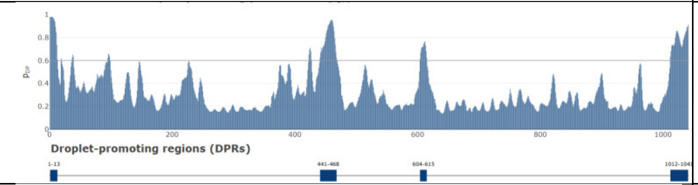	Chicken<br>*Gallus gallus*<br>NP_001026359	1041	30.84%	0.2880	1:1 ortholog
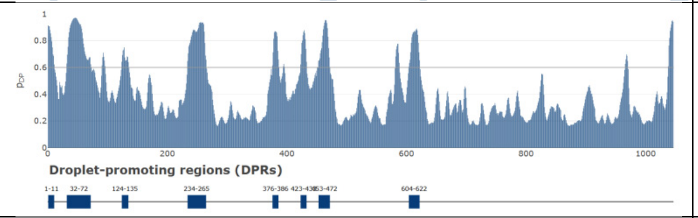	Platypus<br>*Ornithorhynchus anatinus* (2)<br>NP_001229683.1	1046	35.85%	0.4984	1:1 ortholog
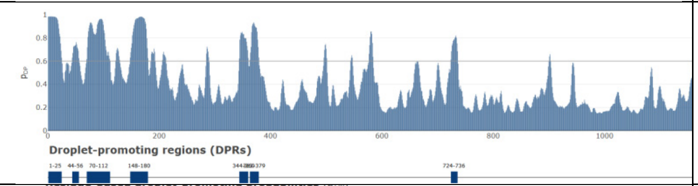	Opossum<br>*Monodelphis domestica*<br>XP_056659760	1162	37.61%	0.6717	1:1 ortholog
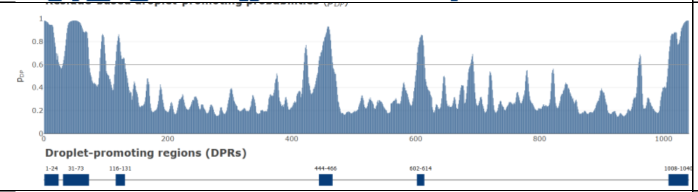	House mouse<br>*Mus musculus*<br>NP_033045	1040	33.17%	0.4973	1:1 ortholog
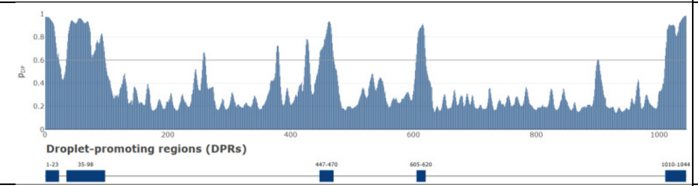	Naked mole rat<br>*Heterocephalus glaber*<br>XP_004852072	1044	34.77%	0.5319	1:1 ortholog
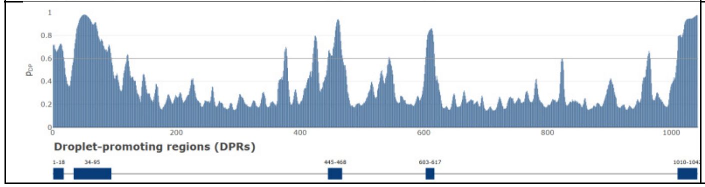	Horse<br>*Equus caballus*<br>NP_001243830	1042	35.32%	0.4330	1:1 ortholog

p_{DP} profiles and DPRs of RAG1 orthologs	Organism<br>Accession No	Sequence length, aa	Overal Disorder	p_{LLPS} score	Relation to human RAG1
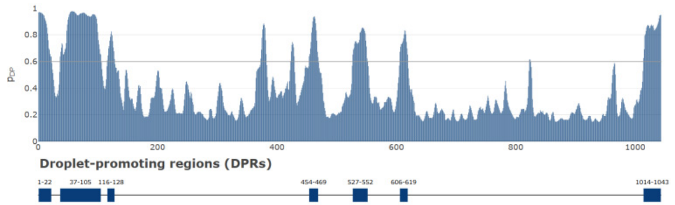	Domestic cat<br>*Felis catus*<br>XP_019667845.1	1043	34.04%	0.5578	1:1 ortholog
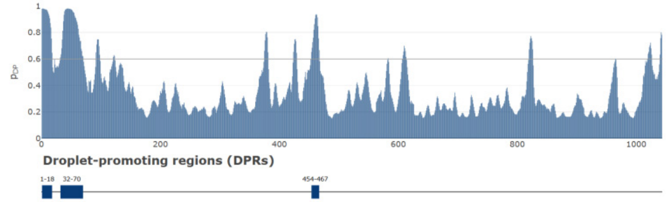	Indian elephant<br>*Elephas maximus*<br>XP_049745708.1	1043	33.94%	0.2728	1:1 ortholog
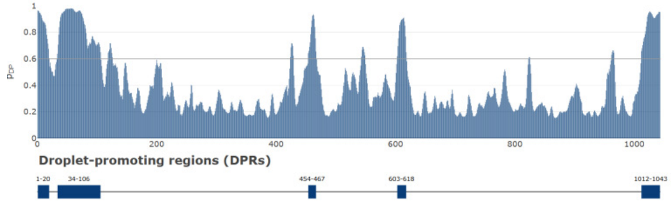	Blue whale<br>*Balaenoptera musculus*<br>XP_036718106.1	1043	34.52%	0.6596	1:1 ortholog
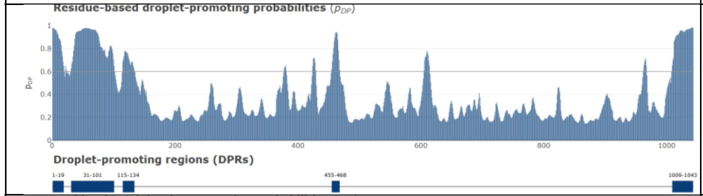	Marmoset<br>*Callithrix jacchus*<br>XP_009006192	1043	32.31%	0.5333	1:1 ortholog
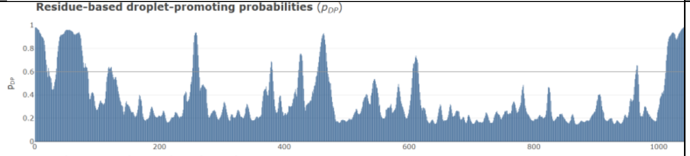	Rhesus monkey<br>*Macaca mulatta*<br>NP_001253701	1043	34.90%	0.4441	1:1 ortholog
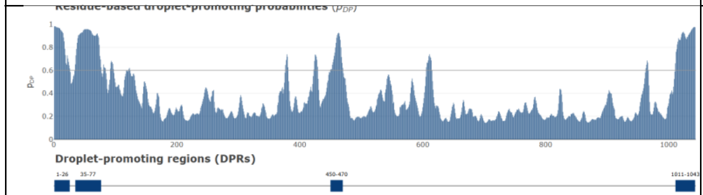	Western lowland gorilla<br>*Gorilla gorilla gorilla*<br>*XP_055211515.2*	1043	32.02%	0.5850	1:1 ortholog

<table>
<tr><th>p_{DP} profiles and DPRs of RAG1 orthologs</th><th>Organism
Accession No</th><th>Sequence length, aa</th><th>Overal Disorder</th><th>p_{LLPS} score</th><th>Relation to human RAG1</th></tr>
<tr><td>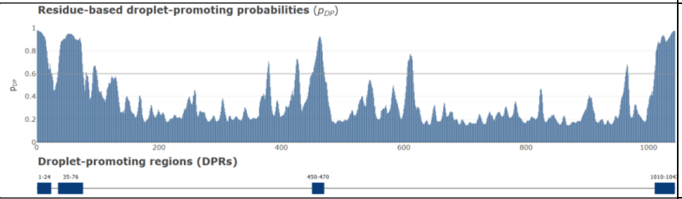
</td><td>Chimp
Pan troglodytes
XP_001154240</td><td>1043</td><td>34.13%</td><td>0.5172</td><td>1:1 ortholog</td></tr>
<tr><td>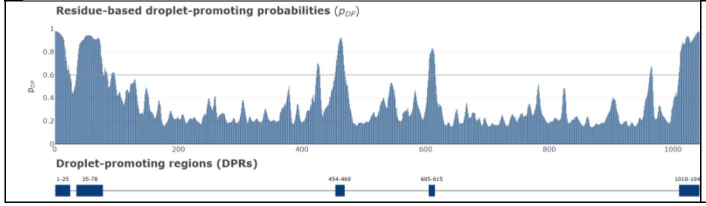
</td><td>Human
Homo sapiens
NP_000439</td><td>1043</td><td>32.89%</td><td>0.5714</td><td></td></tr>
</table>

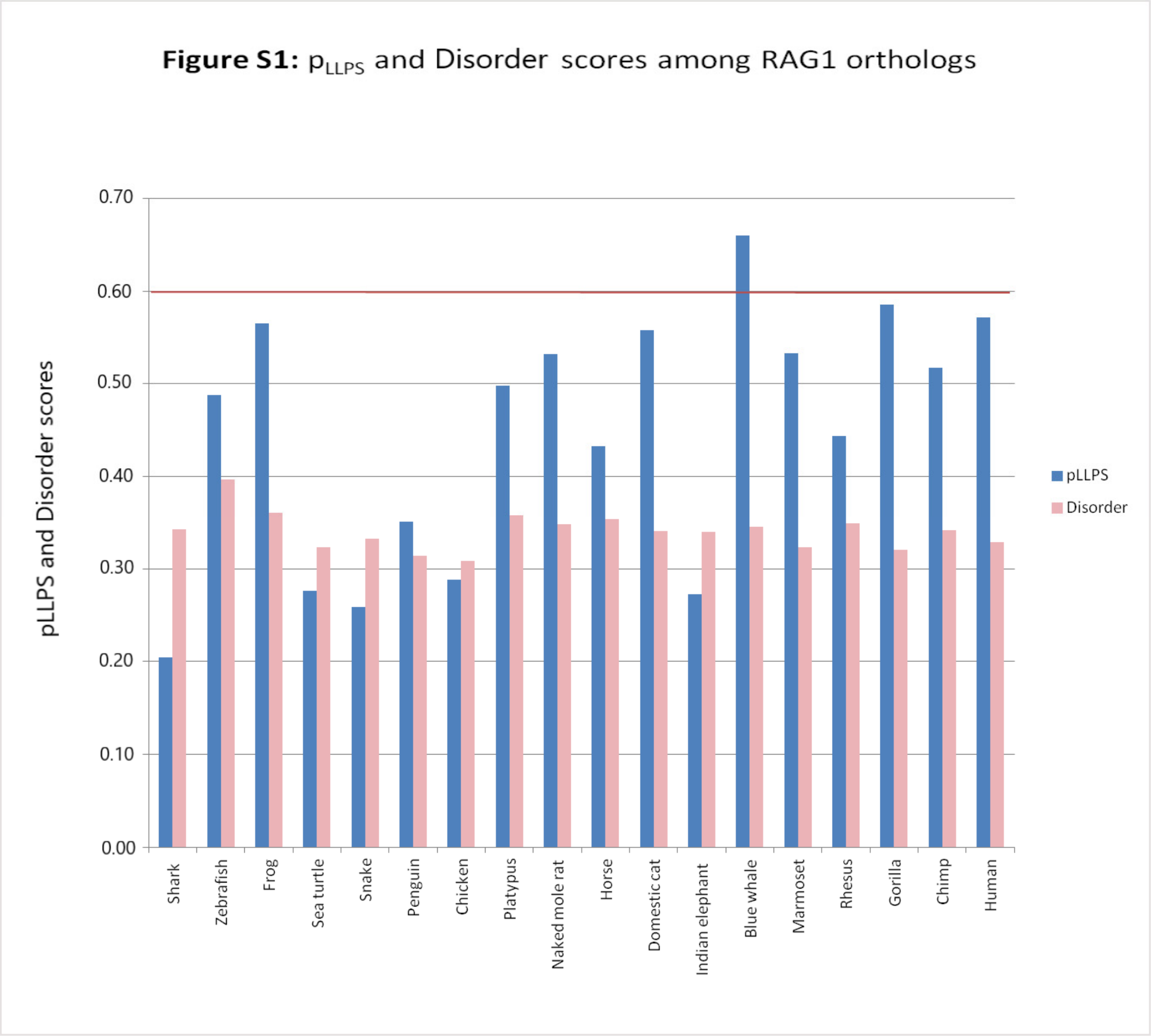

Figure S1. Predicted pLLPS and disorder scores among RAG1 orthologs. Comparative analysis of the phase separation propensity (p_{LLPS}, blue bars) and overall structural disorder (pink bars) for RAG1 protein sequences across 18 vertebrate species. The red horizontal line indicates the p_{LLPS} threshold of 0.60, above which proteins are considered to have a high propensity for liquid-liquid phase separation (LLPS). The data demonstrates that while disorder levels remain relatively stable across taxa, the pLLPS potential shows significant evolutionary variation, with the highest score observed in *Balaenoptera musculus* (Blue whale) and near-threshold value in most mammals. See main text (Section 6.2.) for further details.

Supplementary Table S4. Droplet-promoting probability, Disorder scores, Phase separation propensity and Aggregation potential of **Arc** orthologs and related retrotransposon-derived sequences

p_{DP} profiles, DPRs & Aggregation Hot-spots	Organism Accession No.	Length, aa	Overal Disorder	p_{LLPS} Score	Amyloidogenic Regions (#)	Free Energy (kcal/mol)	Relation to human Arc, Notes
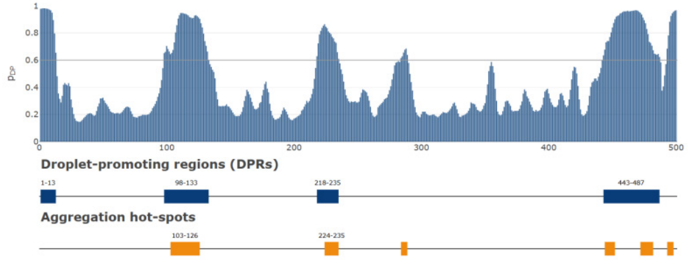	Pr55(Gag) (Human immunodeficiency virus 1) NP_057850.1	500	52.4%	0.4511	41	-7.640	Distant structural homolog; viral origin
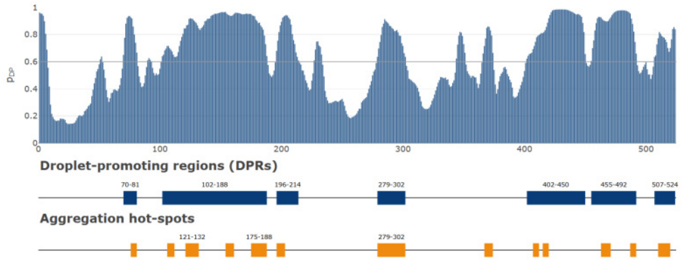	gag protein [Porcine endogenous retrovirus] AAP21815.1	524	55.73%	**0.9584**	40	-9.201	Viral Gag-homolog; non-exapted endogenous retrovirus
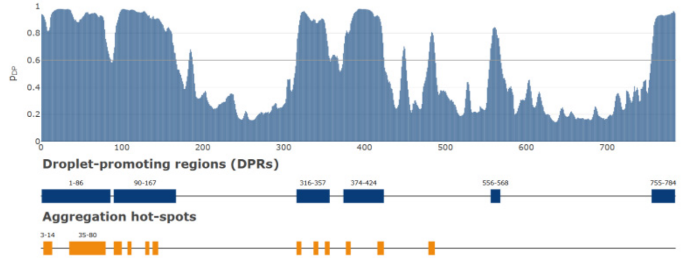	*Homo sapiens* PEG10 NP_001165908.1	784	48.98%	0.8856	26	-5.843	Exapted retroviral Gag-like protein; placental development
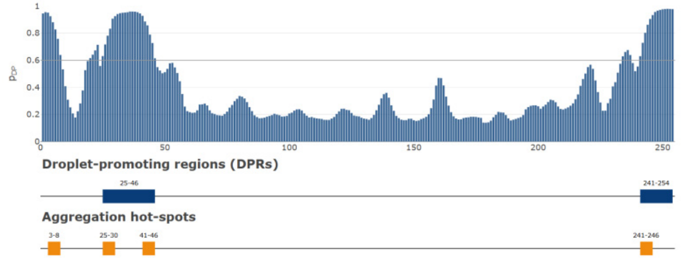	Fruit fly *Drosophila* dArc1 *melanogaster* NP_610955.1	254	37.80%	0.2582	1	-5.432	Functional homolog (Gag-like); convergent retrotransposon domestication

p_{DP} profiles, DPRs & Aggregation Hot-spots	Organism<br>Accession No.	Length, aa	Overal Disorder	p_{LLPS} Score	Amyloidogenic Regions (#)	Free Energy (kcal/mol)	Relation to human Arc, Notes
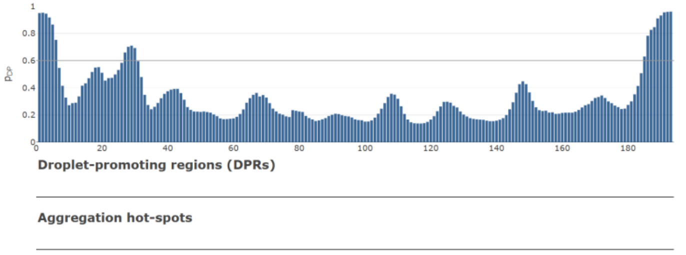	Fruit fly<br>*Drosophila melanogaster*<br>dArc 2 (1)<br>NP_610956.1	193	30.05%	0.2023	30	-7.680	Functional homolog (Gag-like); convergent retrotransposon domestication
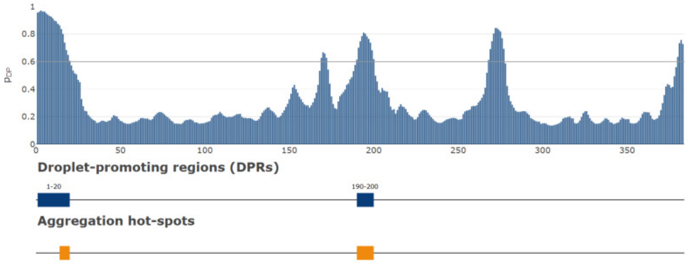	Tropical clawed frog<br>*Xenopus tropicalis* (1)<br>XP_004918984	383	34.46%	0.1923	40	-9.462	1:1 ortholog
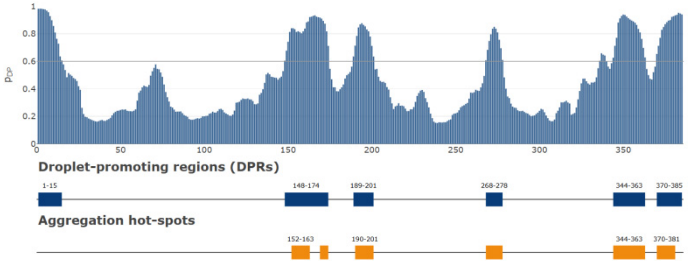	Fire-bellied toad<br>*Bombina bombina* (1)<br>XP_053569189.1	385	42.08%	0.6620	54	-9.462	1:1 ortholog
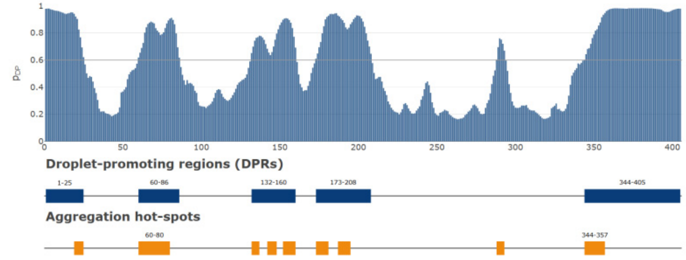	Green sea turtle<br>*Chelonia mydas*<br>XP_027676660	405	50.12%	0.9248	7	-6.954	1:1 ortholog

p_{DP} profiles, DPRs & Aggregation Hot-spots	Organism Accession No.	Length, aa	Overal Disorder	p_{LLPS} Score	Amyloidogenic Regions (#)	Free Energy (kcal/mol)	Relation to human Arc, Notes
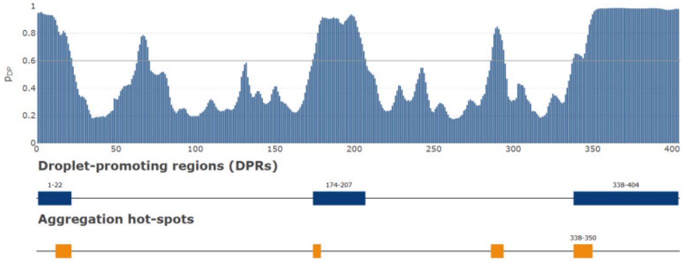	Alligator *Alligator mississippiensis* (1) XP_019337372.1	404	46.04%	0.6625	9	-6.953	1:1 ortholog
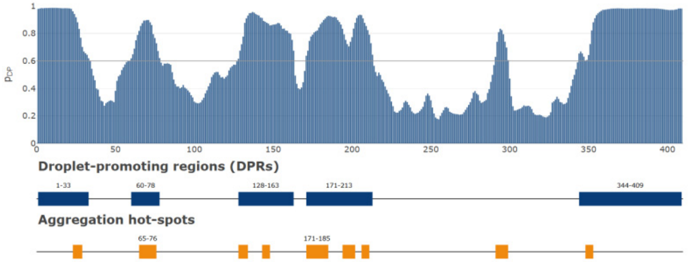	Snake (adder) *Vipera berus* (1) XP_081182022.1	409	44.01%	0.9521	7	-6.985	1:1 ortholog
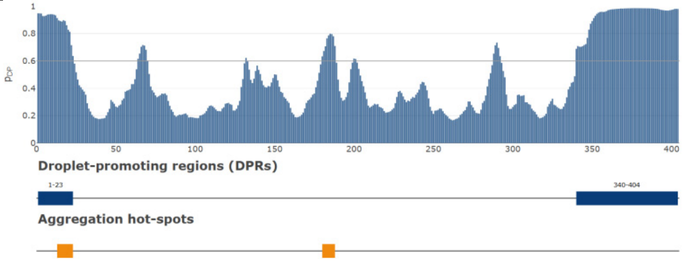	Chicken *Gallus gallus* (1) NP_989763	404	42.33%	0.5072	7	-6.954	1:1 ortholog
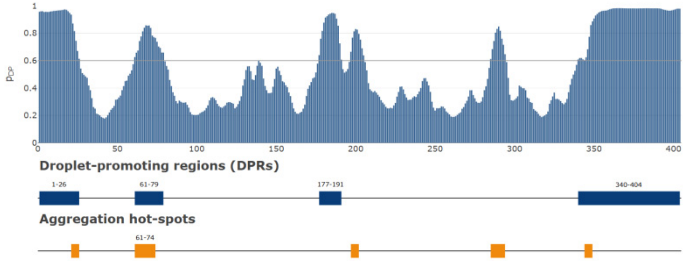	New Caledonian crow *Corvus moneduloides* (1) XP_031947489.1	404	42.82%	0.7434	7	-6.954	1:1 ortholog

p_{DP} profiles, DPRs & Aggregation Hot-spots	Organism<br>Accession No.	Length, aa	Overal Disorder	p_{LLPS} Score	Amyloidogenic Regions (#)	Free Energy (kcal/mol)	Relation to human Arc, Notes
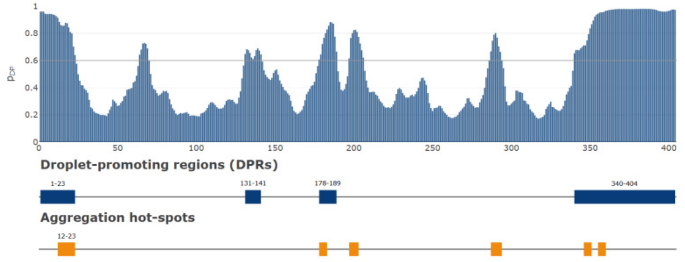	Tawny owl<br>*Strix aluco* (1)<br>XP_074702458.1	404	43.32%	0.5758	7	-6.954	1:1 ortholog
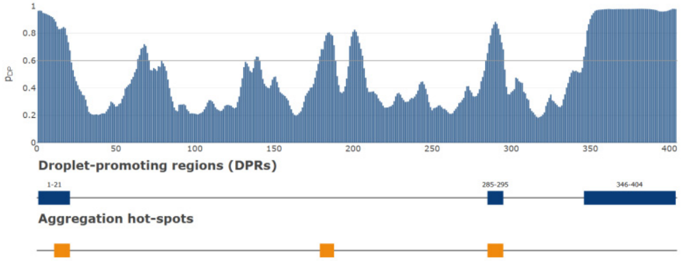	Emperor penguin<br>*Aptenodytes forsteri* (1)<br>XP_009280437.1	404	43.56%	0.6253	7	-6.954	1:1 ortholog
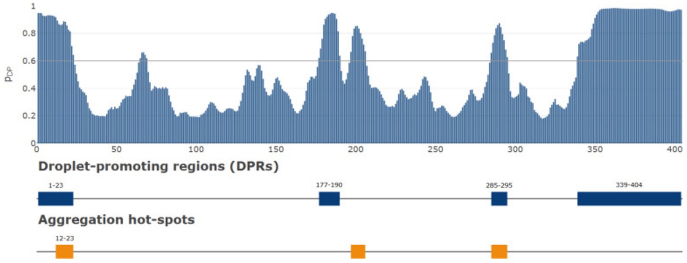	Anna's hummingbird<br>*Calypte anna* (1)<br>XP_030324644.1	404	43.32%	0.5329	7	-6.954	1:1 ortholog
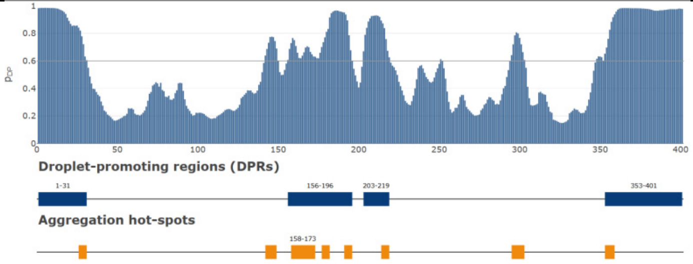	Platypus<br>*Ornithorhynchus anatinus* (1)<br>XP_001512750.1	401	51.37%	0.7980	8	-6.954	1:1 ortholog
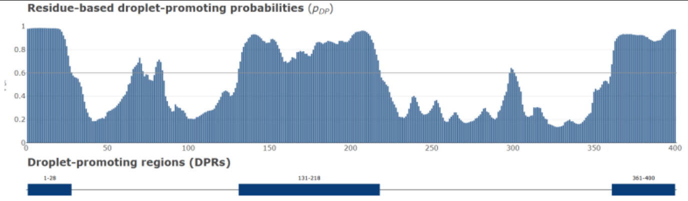	Opossum<br>*Monodelphis domestica*<br>XP_007488477	400	55.25%	0.9179	8	-6.954	1:1 ortholog

p_{DP} profiles, DPRs & Aggregation Hot-spots	Organism<br>Accession No.	Length, aa	Overal Disorder	p_{LLPS} Score	Amyloidogenic Regions (#)	Free Energy (kcal/mol)	Relation to human Arc, Notes
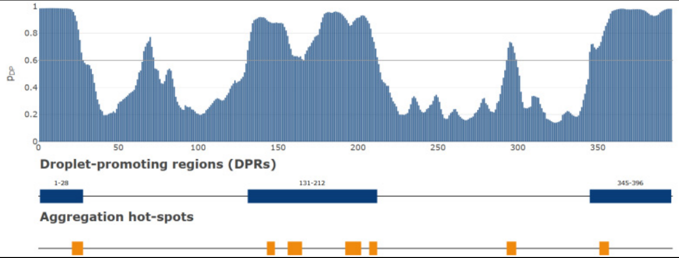	Koala<br>*Phascolarctos cinereus* (1)<br>XP_020851057.1	396	54.55%	0.9179	8	-6.954	1:1 ortholog
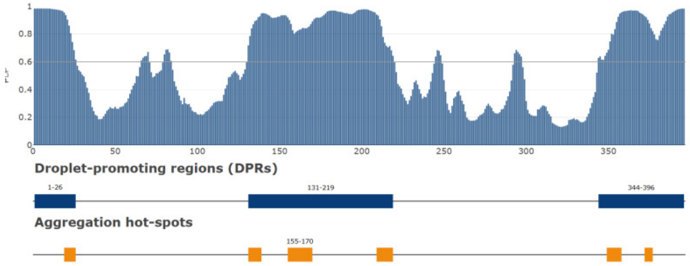	House mouse<br>*Mus musculus* (2)<br>NP_061260.1	396	55.3%	0.9243	38	-6.954	1:1 ortholog
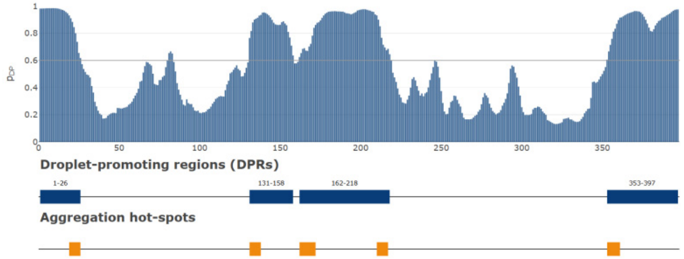	Naked mole rat<br>*Heterocephalus glaber* (1)<br>XP_004837932.1	397	55.16%	0.8877	38	-6.954	1:1 ortholog
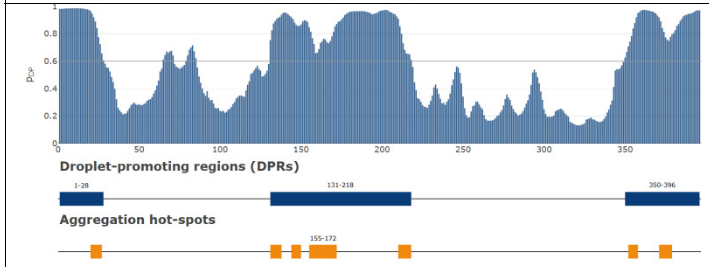	Domestic cat<br>*Felis catus* (1)<br>XP_023104327.1	396	55.81%	0.9243	38	-6.954	1:1 ortholog

p_{DP} profiles, DPRs & Aggregation Hot-spots	Organism<br>Accession No.	Length, aa	Overal Disorder	p_{LLPS} Score	Amyloidogenic Regions (#)	Free Energy (kcal/mol)	Relation to human Arc, Notes
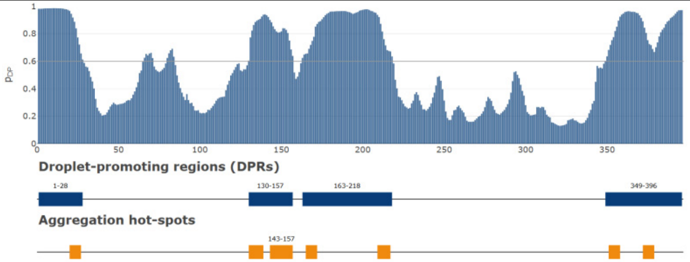	Lion<br>*Panthera leo* (1)<br>XP_042779550.1	396	55.81%	0.9310	38	-6.954	1:1 ortholog
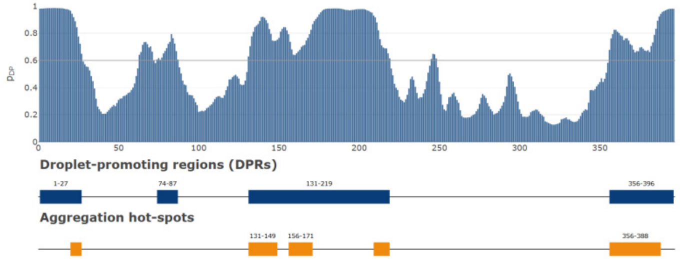	Domestic dog<br>*Canis lupus familiaris* (3)<br>XP_038282315.1	396	58,84%	0.9024	38	-6.954	1:1 ortholog
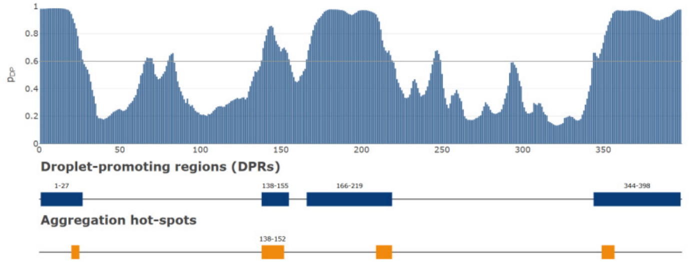	Horse<br>*Equus caballus* (1)<br>XP_014583603	398	55.53%	0.8979	27	-6.954	1:1 ortholog
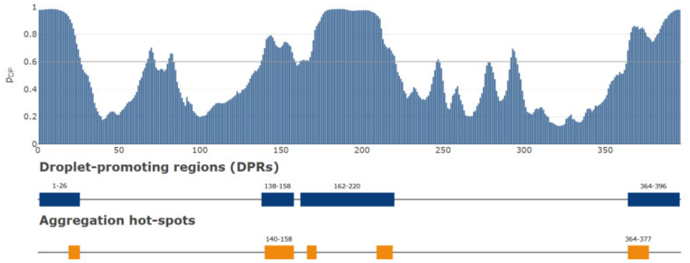	Asian elephant<br>*Elephas maximus indicus* (1)<br>XP_049709834.1	396	54.80%	0.8638	100 (!)	-7.818	1:1 ortholog

p_{DP} profiles, DPRs & Aggregation Hot-spots	Organism<br>Accession No.	Length, aa	Overal Disorder	p_{LLPS} Score	Amyloidogenic Regions (#)	Free Energy (kcal/mol)	Relation to human Arc, Notes
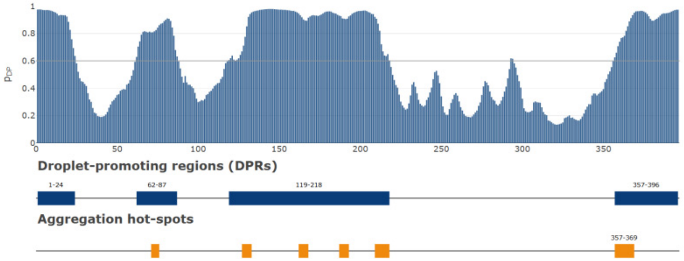	Bottle-nose dolphin<br>*Tursiops truncates* (1)<br>XP_033699013.1	396	57.58%	0.9276	38	-6.954	1:1 ortholog
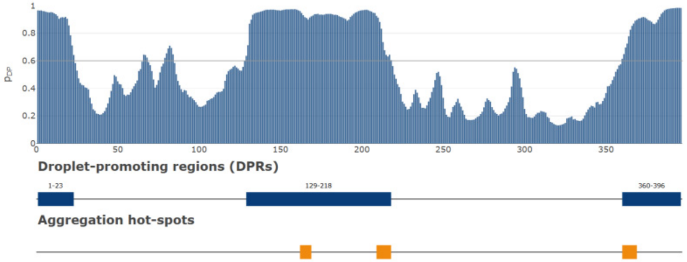	Blue whale<br>*Balaenoptera musculus* (1)<br>XP_036686940.1	396	57.58%	0.8100	38	-6.954	1:1 ortholog
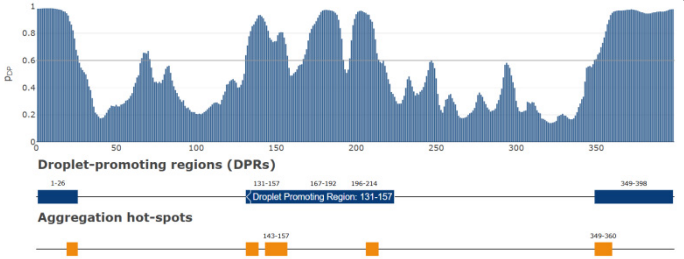	White-tufted-ear marmoset<br>*Callithrix jacchus* (1)<br>XP_035132609.1	398	55.78%	0.8753	38	-6.954	1:1 ortholog
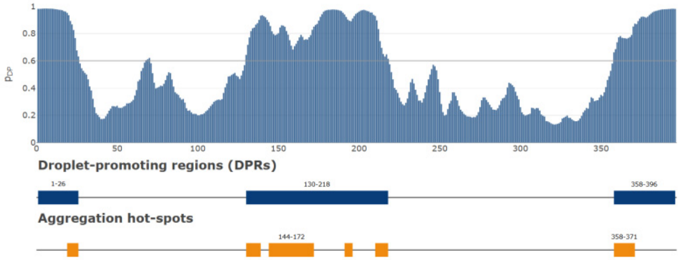	Rhesus monkey<br>*Macaca mulatta*<br>XP_015001586	396	55.81%	0.8110	38	-6.954	1:1 ortholog

p_{DP} profiles, DPRs & Aggregation Hot-spots	Organism<br>Accession No.	Length, aa	Overal Disorder	p_{LLPS} Score	Amyloidogenic Regions (#)	Free Energy (kcal/mol)	Relation to human Arc, Notes
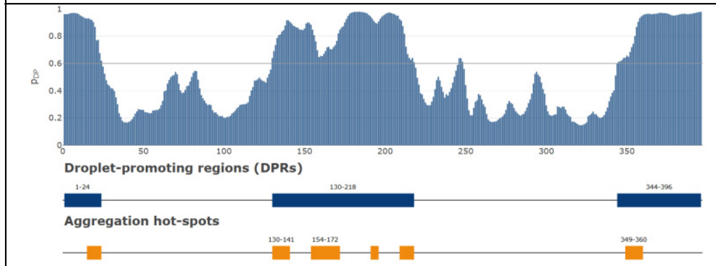	Western lowland gorilla<br>*Gorilla gorilla gorilla* (1)<br>XP_018887452.2	396	55.05%	0.8043	38	-6.954	1:1 ortholog
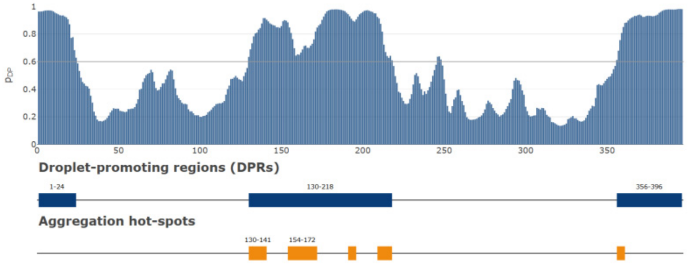	Chimpanzee<br>*Pan troglodytes* (1)<br>XP_016815455	396	55.05%	0.7866	38	-6.954	1:1 ortholog
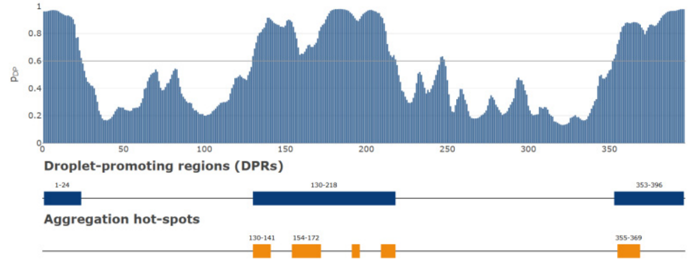	Human<br>*Homo sapiens*<br>NP_056008.1	396	55.05%	0.7943	38	-6.954	

Notes: **Organism / Accession No.** includes vertebrate Arc orthologs, Drosophila Arc isoforms (a case of convergent evolution), and functionally related sequences such as PEG10 and HIV-1 Gag. **Overall Disorder (%)** represents the percentage of residues predicted as disordered by PONDR VL-XT.

pDP Visual Profiles (first column) display the residue-based droplet-promoting probabilities (p_{DP}) as calculated by FuzDrop; blue blocks indicate predicted **Droplet-Promoting Regions (DPRs)**, while orange blocks represent **Aggregation hot-spots**.

Amyloidogenic Regions (#) and the corresponding **Free Energy** (expressed in kcal/mol) were identified using the PASTA 2.0 algorithm to evaluate the propensity for highly ordered beta-sheet aggregation. For further discussion of these regions, see **Main Text (Section 6.3)**.
